# Supplementary material for: Local activity alterations in individuals with autism correlate with neurotransmitter properties and ketamine-induced brain changes
Source: Nat Commun. 2025 Sep 9;16:8248. doi: 10.1038/s41467-025-63857-6 (PMC12420803; doi:10.1038/s41467-025-63857-6)
Supplement: Supplementary file 1 — Supplementary Infomation [file 41467_2025_63857_MOESM1_ESM.pdf]

Supplementary Information for

Local activity alterations in individuals with autism correlate with neurotransmitter properties and ketamine-induced brain changes

Authors: Pascal Grumbach[1,2], Jan Kasper[1,3], Joerg F. Hipp[4], Anna Forsyth[5], Sofie L. Valk[1,3,6,7], Suresh Muthukumaraswamy[5], Simon B. Eickhoff[1,3], Leonhard Schilbach[8,9], Juergen Dukart[1,3*]

Affiliations:

[1]Institute of Neurosciences and Medicine, Brain & Behaviour (INM-7), Research Centre Juelich; Wilhelm-Johnen-Straße 1, 52425 Juelich, Germany.

[2]Department of Psychiatry and Psychotherapy, Medical Faculty and University Hospital Duesseldorf, Heinrich Heine University Duesseldorf; Bergische Landstraße 2, 40629 Duesseldorf, Germany.

[3]Institute of Systems Neuroscience, Medical Faculty & University Hospital Düsseldorf, Heinrich Heine University Düsseldorf; Moorenstraße 5, 40225 Düsseldorf, Germany.

[4]Roche Pharma Research and Early Development, Neuroscience and Rare Diseases, Roche Innovation Center Basel, F. Hoffmann–La Roche Ltd.; Basel, Switzerland.

[5]School of Pharmacy, Faculty of Medical and Health Sciences, University of Auckland; 85 Park Road, Grafton, Auckland, 1023, New Zealand.

[6]Max Planck School of Cognition; Stephanstraße 1A, 04103 Leipzig, Germany.

[7]Max Planck Institute for Human Cognitive and Brain Sciences; Stephanstraße 1A, 04103 Leipzig, Germany.

[8]Department of General Psychiatry 2, LVR-Klinikum Düsseldorf; Bergische Landstraße 2, 40629 Duesseldorf, Germany.

[9]Department of Psychiatry and Psychotherapy, University Hospital, Ludwig Maximilians University Munich; Nußbaumstraße 7, 80336 München, Germany.

*Corresponding author. Email: j.dukart@fz-juelich.de

Results

Supplementary Tables S1-4. Local functional activity alterations in autism compared to TD

We found a replicable pattern of local synchronization (LCOR) reductions in individuals with autism in the default mode network (DMN), anterior cingulate cortex, paracingulate gyrus, precentral gyrus and right insular and opercular cortex: Increased LCOR in autism was found in bilateral temporal regions, the cerebellum, right angular gyrus and lateral occipital cortex:

contrast	cluster (x, y, z)	size	peaks	TFCE	peak *p-FWE*	peak *p-FDR*
**autism >TD**	-45 +00 -24	67	4	313.04	.0030	.0044
	+51 -12 -18	87	7	220.45	.0100	.0070
	-33 -21 -15	12	2	201.43	.0150	.0070
	+51 -54 +12	14	1	185.57	.0220	.0070
	-42 -36 -30	10	1	182.86	.0300	.0070
	-33 +03 -45	14	4	177.16	.0350	.0070
	-27 -15 -27	6	1	175.80	.0390	.0070
	+27 +06 -39	3	1	175.59	.0400	.0070
	+48 -63 +18	6	1	173.68	.0420	.0070
	+60 -48 +09	5	1	172.69	.0430	.0070
	-36 -12 -27	2	1	169.24	.0440	.0070
	+57 -39 +00	5	1	168.51	.0440	.0070
	-45 -30 -30	5	1	167.35	.0460	.0070
	-51 -33 -03	7	1	166.27	.0460	.0070
	-51-39 +06	3	1	166.06	.0460	.0070
	-36 -39 -21	1	1	165.07	.0470	.0070
	-30 -03 -45	1	1	163.23	.0490	.0070
**autism <TD**	-03 -51 +21	491	15	-596.38	< .0001	< .0001
	+00 -21 +06	151	7	-368.78	< .0001	< .0001
	+45 +09 -06	47	2	-236.67	.0040	.0007
	-09 +54 -09	72	7	-225.19	.0050	.0011
	+00 +42 -06	29	3	-190.17	.0170	.0027
	-06 +57 +09	23	3	-185.78	.0220	.0029
	-03 +12 +27	15	2	-170.32	.0340	.0040

Supplementary Table S1. Voxel-wise comparisons (*t*-tests) between autism (n = 405) and TD (n = 473) regarding LCOR in the ABIDE1 cohort. List of significant TFCE-corrected cluster for positive (autism>TD) and negative contrast (autism<TD); highlighted cluster coordinates indicate the voxel with the highest T-value within a cluster; ABIDE = autism brain imaging data exchange; TD = typically developed controls; TFCE = threshold free cluster enhancement; FDR = false discovery rate; FWE = family-wise error; LCOR = local synchronization; TFCE-threshold: $p < 0.05$. Voxel-wise NIfTI files are available from the public repository (reference).

Contrast: autism>TD
28 voxels (11%) covering 4% of atlas.TP l (Temporal Pole Left)
23 voxels (9%) covering 18% of atlas.aMTG l (Middle Temporal Gyrus, anterior division Left)
22 voxels (9%) covering 3% of atlas.TP r (Temporal Pole Right)
19 voxels (8%) covering 23% of atlas.aSTG r (Superior Temporal Gyrus, anterior division Right)
13 voxels (5%) covering 10% of atlas.aMTG r (Middle Temporal Gyrus, anterior division Right)
13 voxels (5%) covering 3% of atlas.pMTG r (Middle Temporal Gyrus, posterior division Right)
12 voxels (5%) covering 5% of atlas.Hippocampus l
11 voxels (4%) covering 4% of atlas.pTFusC l (Temporal Fusiform Cortex, posterior division Left)
8 voxels (3%) covering 2% of atlas.toMTG r (Middle Temporal Gyrus, temporooccipital part Right)
7 voxels (3%) covering 8% of atlas.aSTG l (Superior Temporal Gyrus, anterior division Left)
7 voxels (3%) covering 2% of atlas.pMTG l (Middle Temporal Gyrus, posterior division Left)
6 voxels (2%) covering 0% of atlas.sLOC r (Lateral Occipital Cortex, superior division Right)
6 voxels (2%) covering 6% of atlas.aTFusC l (Temporal Fusiform Cortex, anterior division Left)
5 voxels (2%) covering 4% of atlas.pSTG l (Superior Temporal Gyrus, posterior division Left)
5 voxels (2%) covering 1% of atlas.AG r (Angular Gyrus Right)
4 voxels (2%) covering 1% of atlas.pITG l (Inferior Temporal Gyrus, posterior division Left)
2 voxels (1%) covering 2% of atlas.pSTG r (Superior Temporal Gyrus, posterior division Right)
1 voxels (0%) covering 1% of atlas.aITG l (Inferior Temporal Gyrus, anterior division Left)
1 voxels (0%) covering 1% of atlas.aPaHC r (Parahippocampal Gyrus, anterior division Right)
1 voxels (0%) covering 1% of atlas.PP r (Planum Polare Right)
1 voxels (0%) covering 0% of atlas.Cereb6 l (Cerebelum 6 Left)
53 voxels (21%) covering 0% of atlas.not-labeled
Contrast: autism<TD
323 voxels (39%) covering 45% of atlas.PC (Cingulate Gyrus, posterior division)
136 voxels (16%) covering 8% of atlas.Precuneous (Precuneous Cortex)
83 voxels (10%) covering 20% of atlas.Thalamus l
63 voxels (8%) covering 16% of atlas.Thalamus r
31 voxels (4%) covering 8% of atlas.IC r (Insular Cortex Right)
29 voxels (4%) covering 1% of atlas.FP l (Frontal Pole Left)
28 voxels (3%) covering 4% of atlas.AC (Cingulate Gyrus, anterior division)
24 voxels (3%) covering 1% of atlas.FP r (Frontal Pole Right)
21 voxels (3%) covering 6% of atlas.PaCiG l (Paracingulate Gyrus Left)
19 voxels (2%) covering 7% of atlas.MedFC (Frontal Medial Cortex)
9 voxels (1%) covering 2% of atlas.PaCiG r (Paracingulate Gyrus Right)
5 voxels (1%) covering 1% of atlas.TP r (Temporal Pole Right)
3 voxels (0%) covering 0% of atlas.PreCG l (Precentral Gyrus Left)
2 voxels (0%) covering 1% of atlas.IFG oper r (Inferior Frontal Gyrus, pars opercularis Right)
1 voxels (0%) covering 0% of atlas.PreCG r (Precentral Gyrus Right)
1 voxels (0%) covering 1% of atlas.FO r (Frontal Operculum Cortex Right)
1 voxels (0%) covering 0% of atlas.CO r (Central Opercular Cortex Right)
49 voxels (6%) covering 0% of atlas.not-labeled

Supplementary Table S2. Regions with significant voxels in ABIDE1 (all clusters combined). List of all significant voxels and atlas regions in the autism brain imaging data exchange (ABIDE) 1 dataset for the positive (autism>TD) and negative contrast (autism<TD); n = 405 autism, n = 473 TD; TD = typically developed controls. Voxel-wise NIfTI files are available from the public repository (reference).

contrast	cluster (x, y, z)	size	peaks	TFCE	peak *p-FWE*	peak *p-FDR*
**autism >TD**	-06 -39 -60	46	4	-186.59	.0220	.0157
	-39 -63 +09	10	1	-183.92	.0240	.0157
	+06 -03 +60	13	2	-176.54	.0280	.0157
	+18 -48 -57	11	3	-172.64	.0320	.0157
	-21 +15 -42	7	2	-171.96	.0320	.0157
	-18 -39 -54	4	1	-167.94	.0370	.0157
	+30 +12 -42	14	1	-166.09	.0390	.0157
	-36 -57 -60	6	1	-165.65	.0400	.0157
	-21 +06 -45	5	2	-163.34	.0410	.0157
	+42 +06 -45	5	1	-160.92	.0460	.0157
	+30 -48 -57	4	1	-160.61	.0470	.0157
	+45 -48 +45	4	1	-160.45	.0470	.0157
	+39 -06 -42	8	1	-159.69	.0470	.0157
**autism <TD**	-03 +48 +00	306	15	333.74	< .0001	< .0001
	+09 -48 +30	98	8	330.19	< .0001	< .0001
	+63 -09 +30	480	41	316.08	< .0001	< .0001
	+66 -12 +00	4	1	167.70	.0380	.0030
	-63 -03 +24	9	1	163.00	.0470	.0035
	+27 +27 -03	1	1	160.93	.0500	.0038

Supplementary Table S3. Voxel-wise comparisons (*t*-tests) between autism (n = 395) and TD (n = 474) regarding LCOR in the ABIDE2 cohort. List of significant TFCE-corrected cluster for positive (autism>TD) and negative contrast (autism<TD); highlighted cluster coordinates indicate the voxel with the highest T-value within a cluster; ABIDE = autism brain imaging data exchange; TD = typically developed controls; TFCE = threshold free cluster enhancement; FDR = false discovery rate; FWE = family-wise error; LCOR = local synchronization; TFCE-threshold: $p < 0.05$. Voxel-wise NIfTI files are available from the public repository (reference).

Contrast: autism>TD
42 voxels (31%) covering 3% of atlas.Brain-Stem
14 voxels (10%) covering 2% of atlas.TP r (Temporal Pole Right)
13 voxels (9%) covering 7% of atlas.SMA r (Supplementary Motor Cortex- Right)
12 voxels (9%) covering 2% of atlas.Cereb8 r (Cerebelum 8 Right)
11 voxels (8%) covering 2% of atlas.TP l (Temporal Pole Left)
6 voxels (4%) covering 6% of atlas.aITG r (Inferior Temporal Gyrus, anterior division Right)
5 voxels (4%) covering 2% of atlas.Cereb9 l (Cerebelum 9 Left)
3 voxels (2%) covering 1% of atlas.pSMG r (Supramarginal Gyrus, posterior division Right)
3 voxels (2%) covering 1% of atlas.Cereb8 l (Cerebelum 8 Left)
2 voxels (1%) covering 2% of atlas.aTFusC r (Temporal Fusiform Cortex, anterior division Right)
2 voxels (1%) covering 1% of atlas.pTFusC r (Temporal Fusiform Cortex, posterior division Right)
1 voxels (1%) covering 0% of atlas.toMTG l (Middle Temporal Gyrus, temporooccipital part Left)
1 voxels (1%) covering 0% of atlas.pITG r (Inferior Temporal Gyrus, posterior division Right)
1 voxels (1%) covering 0% of atlas.AG r (Angular Gyrus Right)
21 voxels (15%) covering 0% of atlas.not-labeled
Contrast: autism<TD
105 voxels (12%) covering 26% of atlas.IC r (Insular Cortex Right)
100 voxels (11%) covering 10% of atlas.PostCG r (Postcentral Gyrus Right)
83 voxels (9%) covering 12% of atlas.PC (Cingulate Gyrus, posterior division)
76 voxels (8%) covering 6% of atlas.PreCG r (Precentral Gyrus Right)
72 voxels (8%) covering 19% of atlas.PaCiG r (Paracingulate Gyrus Right)
62 voxels (7%) covering 3% of atlas.FP r (Frontal Pole Right)
62 voxels (7%) covering 17% of atlas.PaCiG l (Paracingulate Gyrus Left)
56 voxels (6%) covering 21% of atlas.CO r (Central Opercular Cortex Right)
47 voxels (5%) covering 6% of atlas.AC (Cingulate Gyrus, anterior division)
28 voxels (3%) covering 12% of atlas.aSMG r (Supramarginal Gyrus, anterior division Right)
26 voxels (3%) covering 1% of atlas.FP l (Frontal Pole Left)
25 voxels (3%) covering 16% of atlas.PO r (Parietal Operculum Cortex Right)
17 voxels (2%) covering 6% of atlas.MedFC (Frontal Medial Cortex)
15 voxels (2%) covering 3% of atlas.FOrb r (Frontal Orbital Cortex Right)
13 voxels (1%) covering 1% of atlas.Precuneous (Precuneous Cortex)
12 voxels (1%) covering 9% of atlas.PT r (Planum Temporale Right)
11 voxels (1%) covering 12% of atlas.FO r (Frontal Operculum Cortex Right)
9 voxels (1%) covering 4% of atlas.IFG oper r (Inferior Frontal Gyrus, pars opercularis Right)
4 voxels (0%) covering 0% of atlas.PreCG l (Precentral Gyrus Left)
4 voxels (0%) covering 0% of atlas.PostCG l (Postcentral Gyrus Left)
3 voxels (0%) covering 0% of atlas.TP r (Temporal Pole Right)
2 voxels (0%) covering 2% of atlas.pSTG r (Superior Temporal Gyrus, posterior division Right)
2 voxels (0%) covering 2% of atlas.HG r (Heschl's Gyrus Right)
2 voxels (0%) covering 1% of atlas.Putamen r
62 voxels (7%) covering 0% of atlas.not-labeled

Supplementary Table S4. Regions with significant voxels in ABIDE2 (all clusters combined). List of all significant voxels and atlas regions in the autism brain imaging data exchange (ABIDE) 1 dataset for the positive (autism>TD) and negative contrast (autism<TD); n = 395 autism; n = 474 TD; TD = typically developed controls. Voxel-wise NIfTI files are available from the public repository (reference).

Supplementary Figures S1-2. Voxel-wise differences in LCOR between autism and TD controls after changes in preprocessing pipeline

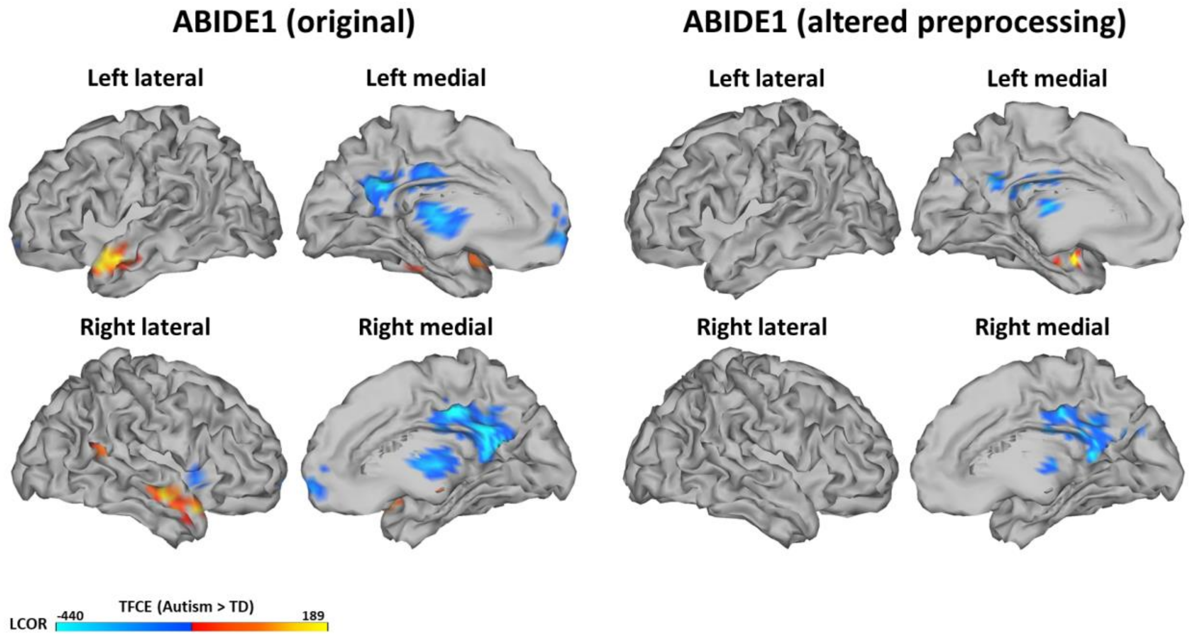

Supplementary Figure S1. Results of voxel-wise two-sided t-tests between autism (n = 405) and TD controls (n = 473) in the ABIDE1 dataset. Left: Original results from the manuscript. Right: Results after removing the first four initial scans, smoothing and gray matter signal regression from preprocessing pipeline. Voxel-wise NIfTI files are available from the public repository (reference). Exact p-values are shown in Supplementary Tables S1-S2.

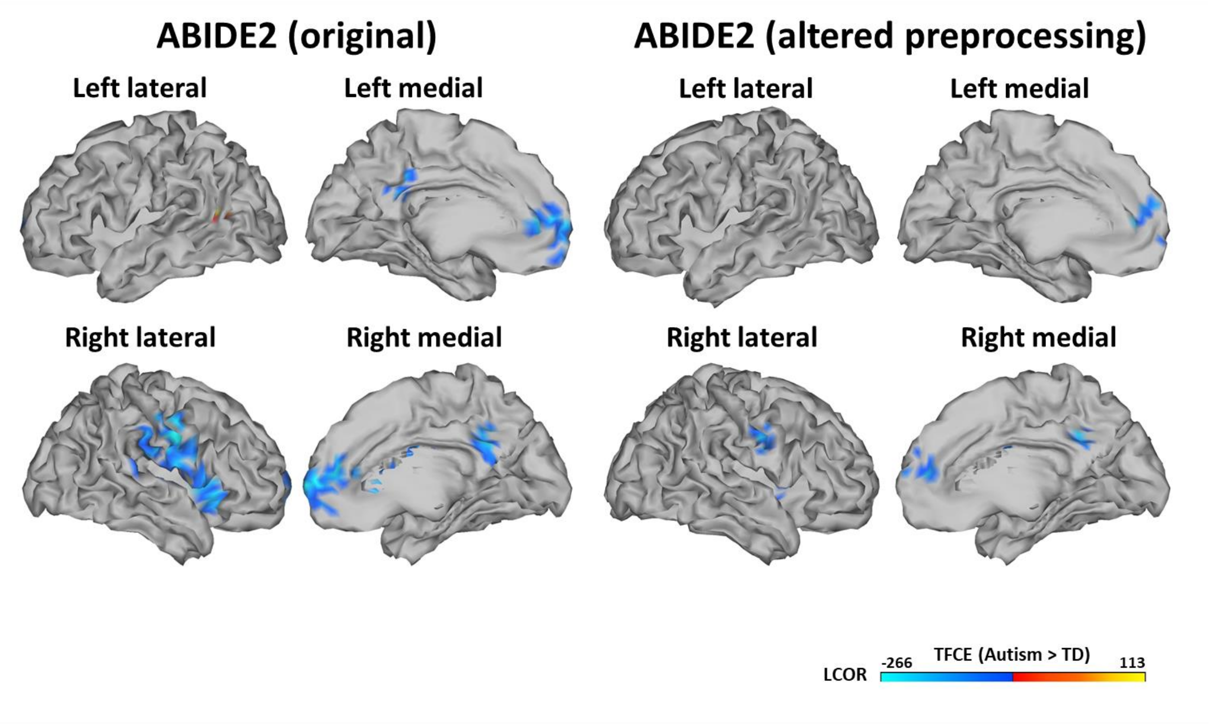

Supplementary Figure S2. Results of voxel-wise two-sided t-tests between autism (n = 395) and TD controls (n = 474) in the ABIDE2 dataset. Left: Original results from the manuscript. Right: Results after removing the first four initial scans, smoothing and gray matter signal regression from preprocessing pipeline. Voxel-wise NIfTI files are available from the public repository (reference). Exact p-values are shown in Supplementary Tables S3-S4.

Supplementary Table S5. LCOR alterations in autism relate to the *in-vivo* distribution of neurotransmitter systems

In ABIDE1, we found significant co-localizations between LCOR alterations in autism compared to TD and serotonergic 5HT4, dopaminergic D1, D2, DAT and FDOPA, glutamatergic NMDA and mGLuR5, GABAa, NAT and VAChT distributions from nuclear imaging. The significant co-localizations with D1, D2, DAT, NMDA, GluR5, NMDA and VAChT were replicated in the ABIDE2 dataset.

	**Co-localization**	**ABIDE1**		**ABIDE2**	
		**Spearman *r***	***p*-value**	**Spearman *r***	***p*-value**
**LCOR**	5HT1a	.1982	.1349	.0492	.7213
	5HT1b	-.2480	.0809	-.4308	< .0010***
	5HT2a	.0555	.6993	-.0002	.9980
	5HT4	.1945	.0240*	-.0334	.7423
	SERT	-.0393	.6553	-.0245	.7982
	D1	-.2634	.0050**	-.2778	.0020**
	D2	-.3216	< .0010***	-.3239	< .0010***
	DAT	-.1867	.0330*	-.1963	.0280*
	FDOPA	-.2000	.0340*	-.0794	.3996
	NMDA	-.3318	.0090**	-.3109	.0090**
	mGluR5	-.3786	.0040**	-.3923	.0030**
	GABAa	-.2478	.0050**	-.1864	.0350*
	CB1	-.1307	.3816	-.3348	.0110*
	MU	-.1056	.4905	-.1484	.3400
	NAT	-.2018	.0240*	-.1641	.0639
	VAChT	-.2409	.0030**	-.2378	.0060**

Supplementary Table S5. Statistical data of co-localizations (two-sided) between LCOR alterations in autism (n = 405, n = 395) compared to TD (n = 473, n = 474) and neurotransmitter systems in ABIDE1 and ABIDE2. ABIDE = autism brain imaging data exchange; LCOR = local synchronization; TD = typically developed controls; 5HT = 5-hydroxytryptamine (serotonin receptor); SERT = serotonin transporter; D1/D2 = dopamine receptor; DAT = dopamine transporter; FDOPA = fluorodopa; NMDA = N-Methyl-D-Aspartat receptor; mGluR5 = metabotropic glutamate receptor; GABAa = γ-aminobutyric acid type A receptor; CB1 = cannabinoid receptor; MU = μ-opioid receptor; NAT = noradrenaline transporter; VAChT = vesicular acetylcholine transporter; * = $p < .05$; ** = $p < .01$; *** = $p < .001$; df = 117. Source data are provided as a Source Data file. Voxel-wise NIfTI files are available from the public repository (reference).

Supplementary Tables S6-7. LCOR-neurotransmitter co-localization profiles after changes in preprocessing pipeline

Co-localization	ABIDE1 (original results)		ABIDE1 (without smoothing and GM regression)	
	Spearman *r*	*p*-value	Spearman *r*	*p*-value
5HT1a	.1982	.1349	.0939	.4785
5HT1b	-.2480	.0809	-.2707	.0499*
5HT2a	.0555	.6993	-.0714	.5984
5HT4	.1945	.0240*	.0679	.4665
SERT	-.0393	.6553	-.1304	.1549
D1	-.2634	.0050**	-.2831	.0030**
D2	-.3216	< .0010***	-.3139	< .0010***
DAT	-.1867	.0330*	-.1864	.0470*
FDOPA	-.2000	.0340*	-.1733	.0509
NMDA	-.3318	.0090**	-.3781	.0020**
mGluR5	-.3786	.0040**	-.4149	.0020**
GABAa	-.2478	.0050**	-.2777	.0020**
CB1	-.1307	.3816	-.1675	.2747
MU	-.1056	.4905	-.0864	.5824
NAT	-.2018	.0240*	-.1663	.0709
VAChT	-.2409	.0030**	-.1893	.0410*

Supplementary Table S6. Statistical data of co-localizations (two-sided) between LCOR alterations in autism (n = 405) compared to TD (n = 473) and neurotransmitter systems in ABIDE1 after removing the first four initial scans, smoothing and gray matter signal regression. ABIDE = autism brain imaging data exchange; GM = gray matter; 5HT = 5-hydroxytryptamine (serotonin receptor); SERT = serotonin transporter; D1/D2 = dopamine receptor; DAT = dopamine transporter; FDOPA = fluorodopa; NMDA = N-Methyl-D-Aspartat receptor; mGluR5 = metabotropic glutamate receptor; GABAa = γ-aminobutyric acid type A receptor; CB1 = cannabinoid receptor; MU = μ-opioid receptor; NAT = noradrenaline transporter; VAChT = vesicular acetylcholine transporter; * = $p < .05$; ** = $p < .01$; *** = $p < .001$; df = 117. Source data are provided as a Source Data file. Voxel-wise NIfTI files are available from the public repository (reference).

Co-localization	ABIDE2 (original results)		ABIDE2 (without smoothing and GM regression)	
	**Spearman *r***	***p*-value**	**Spearman *r***	***p*-value**
5HT1a	.0492	.7213	-.0806	.5165
5HT1b	-.4308	< .0010***	-.4054	.0020**
5HT2a	-.0002	.9980	-.1188	.3456
5HT4	-.0334	.7423	-.0195	.7962
SERT	-.0245	.7982	.0162	.7962
D1	-.2778	.0020**	-.2787	.0030**
D2	-.3239	< .0010***	-.2385	.0070**
DAT	-.1963	.0280*	-.1693	.0640
FDOPA	-.0794	.3996	-.0236	.8012
NMDA	-.3109	.0090**	-.2125	.0689
mGluR5	-.3923	.0030**	-.4075	< .0010***
GABAa	-.1864	.0350*	-.3133	< .0010***
CB1	-.3348	.0110*	-.3192	.0110*
MU	-.1484	.3400	-.0671	.6314
NAT	-.1641	.0639	-.1744	.0569
VAChT	-.2378	.0060**	-.0907	.3237

Supplementary Table S7. Statistical data of co-localizations (two-sided) between LCOR alterations in autism (n = 395) compared to TD (n = 474) and neurotransmitter systems in ABIDE2 after removing the first four initial scans, smoothing and gray matter signal regression. ABIDE = autism brain imaging data exchange; GM = gray matter; 5HT = 5-hydroxytryptamine (serotonin receptor); SERT = serotonin transporter; D1/D2 = dopamine receptor; DAT = dopamine transporter; FDOPA = fluorodopa; NMDA = N-Methyl-D-Aspartat receptor; mGluR5 = metabotropic glutamate receptor; GABAa = γ-aminobutyric acid type A receptor; CB1 = cannabinoid receptor; MU = μ-opioid receptor; NAT = noradrenaline transporter; VAChT = vesicular acetylcholine transporter; * = $p < .05$; ** = $p < .01$; *** = $p < .001$; df = 117. Source data are provided as a Source Data file. Voxel-wise NIfTI files are available from the public repository (reference).

Supplementary Table S8. LCOR-neurotransmitter co-localization profiles by using the combined functional Schaefer + Melbourne/Tian atlas

	**ABIDE1 (Neuromorphometrics)**		**ABIDE1 (Schaefer + Melbourne/Tian)**		**ABIDE2 (Neuromorphometrics)**		**ABIDE2 (Schaefer + Melbourne/Tian)**	
	**Spearman r**	**p-value**	**Spearman r**	**p-value**	**Spearman r**	**p-value**	**Spearman r**	**p-value**
5HT1a	.1982	.1349	.2958	.0370*	.0492	.7213	.0666	.6374
5HT1b	-.2480	.0809	-.0418	.7552	-.4308	< .001***	-.2725	.0519
5HT2a	.0555	.6993	.1120	.3946	-.0002	.9980	.0999	.4326
5HT4	.1945	.0240*	.1027	.2857	-.0334	.7423	-.0512	.5984
SERT	-.0393	.6553	-.2443	< .001***	-.0245	.7982	-.1670	.0689
D1	-.2634	.0050**	-.2756	.0020**	-.2778	.0020**	-.1587	.1029
D2	-.3216	< .001***	-.3708	< .001***	-.3239	< .001***	-.2157	.0160*
DAT	-.1867	.0330*	-.3132	.0020**	-.1963	.0280*	-.2361	.0090**
FDOPA	-.2000	.0340*	-.2075	.1259	-.0794	.3996	-.0256	.8581
NMDA	-.3318	.0090**	-.2989	.0170*	-.3109	.0090**	-.1430	.2817
mGluR5	-.3786	.0040**	-.2378	.0120*	-.3923	.0030**	-.1882	.0400*
GABAa	-.2478	.0050**	-.0613	.5315	-.1864	.0350*	.0716	.4446
CB1	-.1307	.3816	.0462	.7483	-.3348	.0110*	-.1775	.1958
MU	-.1056	.4905	-.0184	.9181	-.1484	.3400	-.1387	.3856
NAT	-.2018	.0240*	-.1797	.0569	-.1641	.0639	.0250	.7752
VAChT	-.2409	.0030**	-.2903	.0020**	-.2378	.0060**	-.2772	.0030**

Supplementary Table S8. Statistical data of co-localizations (two-sided) between LCOR alterations in autism (n = 405, n = 395) compared to TD controls (n = 473, n = 474) and neurotransmitter properties using the anatomical Neuromorphometrics atlas and the functional connectivity-based Schaefer + Melbourne/Tian atlas. ABIDE = autism brain imaging data exchange; TD = typically developed controls; 5HT = 5-hydroxytryptamine (serotonin receptor); SERT = serotonin transporter; D1/D2 = dopamine receptor; DAT = dopamine transporter; FDOPA = fluorodopa; NMDA = N-Methyl-D-Aspartat receptor; mGluR5 = metabotropic glutamate receptor; GABAa = γ-aminobutyric acid type A receptor; CB1 = cannabinoid receptor; MU = μ-opioid receptor; NAT = noradrenaline transporter; VAChT = vesicular acetylcholine transporter; * = $p < .05$; ** = $p < .01$; *** = $p < .001$; df (Neuromorphometrics) = 117; df (Schaefer+Melbourne/Tian) = 114. Source data are provided as a Source Data file.

Supplementary Table S9. Results of meta-analytically combined *p*-values using Fisher's method

To increase the robustness of our exploration and replication design, we have adopted a common meta-analytic approach of combining *p*-values from both ABIDE cohorts using the conservative Fisher's method and applied a false-discovery rate correction to the resulting *p*-values. This resulted in 10 out of 16 associations surviving the correction for multiple comparisons including all of the 7 associations that were replicated in the ABIDE2 cohort. In addition, the associations with 5-HT1b, CB1 and NAT would be considered significant in this meta-analytic approach despite not being significant in both datasets.

	***p*-value (ABIDE1)**	***p*-value (ABIDE2)**	***p*-value (meta-analytically)**
5HT1a	0.1349	0.7213	0.3240
5HT1b	0.0809	< 0.001***	0.0008***
5HT2a	0.6993	0.998	0.9489
5HT4	0.024*	0.7423	0.0896
SERT	0.6553	0.7982	0.8620
D1	0.005**	0.002**	0.0001***
D2	< 0.001***	< 0.001***	0.0000***
DAT	0.033*	0.028*	0.0074**
FDOPA	0.034*	0.3996	0.0720
NMDA	0.009**	0.009**	0.0008***
mGluR5	0.004**	0.003**	0.0001***
GABAa	0.005**	0.035*	0.0017**
CB1	0.3816	0.011*	0.0272*
MU	0.4905	0.34	0.4655
NAT	0.024*	0.0639	0.0115*
VAChT	0.003**	0.006**	0.0002***

Supplementary Table S9. Meta-analytically combined *p*-values from co-localization results (two-sided) in both ABIDE cohorts using Fisher's method. ABIDE = Autism Brain Imaging Data Exchange; 5HT = 5-hydroxytryptamine (serotonin receptor); SERT = serotonin transporter; D1/D2 = dopamine receptor; DAT = dopamine transporter; FDOPA = fluorodopa; NMDA = N-Methyl-D-Aspartat receptor; mGluR5 = metabotropic glutamate receptor; GABAa = γ-aminobutyric acid type A receptor; CB1 = cannabinoid receptor; MU

= μ-opioid receptor; NAT = noradrenaline transporter; VAChT = vesicular acetylcholine transporter; * = $p < .05$; ** = $p < .01$; *** = $p < .001$; df = 117. Source data are provided as a Source Data file.

Supplementary Table S10. Neurotransmitter co-localizations with LCOR changes induced by ketamine and midazolam

We computed spatial Spearman correlations between the whole-brain LCOR T-maps induced by ketamine or midazolam and different neurotransmitter distributions. The ketamine effect on LCOR was co-localized with the distribution of D1, NMDA and GABAa receptors after correcting for multiple comparisons. Midazolam displayed significant co-localizations with 5HT2a, SERT, DAT, FDOPA, CB1, NAT and VAChT.

	Co-localization	Ketamine		Midazolam	
		Spearman *r*	*p-FDR*	Spearman *r*	*p-FDR*
**LCOR**	5HT1a	.0487	.8460	-.1613	.2652
	5HT1b	-.3265	.1199	-.2478	.1191
	5HT2a	-.2299	.2420	-.3052	.0466*
	5HT4	-.0487	.7353	-.0824	.4018
	SERT	-.1261	.3149	.4363	< .001***
	D1	-.2829	.0320*	.0960	.3418
	D2	-.0265	.8109	.1757	.1052
	DAT	-.0270	.7752	.4259	.0016**
	FDOPA	-.1336	.3536	.4169	< .001***
	NMDA	-.3380	.0320*	.2254	.1187
	mGluR5	-.2926	.1375	-.1691	.2797
	GABAa	-.2514	.0373*	-.1760	.0962
	CB1	-.2657	.2504	-.4683	.0022**
	MU	-.1732	.5740	-.1212	.5067
	NAT	.0778	.5395	.2639	.0096**
	VAChT	-.1236	.3481	.3324	< .001***

Supplementary Table S10. Statistical data (n = 27) of co-localizations between LCOR alterations induced by ketamine and midazolam compared to placebo and neurotransmitter systems. LCOR = local synchronization; FDR = false discovery rate; 5HT = 5-hydroxytryptamine (serotonin receptor); SERT = serotonin transporter; D1/D2 = dopamine receptor; DAT = dopamine transporter; FDOPA = fluorodopa; NMDA = N-Methyl-D-Aspartat receptor; mGluR5 = metabotropic glutamate receptor; GABAa = γ-aminobutyric acid type A receptor; CB1 = cannabinoid receptor; MU = μ-opioid receptor; NAT = noradrenaline transporter; VAChT = vesicular acetylcholine transporter; CBF = cerebral blood flow; * = $p < .05$; ** = $p < .01$; *** = $p < .001$; df = 117. Source data are provided as a Source Data file. Voxel-wise NIfTI files are available from the public repository (reference).

Supplementary Fig. S3. Visualization of ketamine and midazolam co-localization profiles

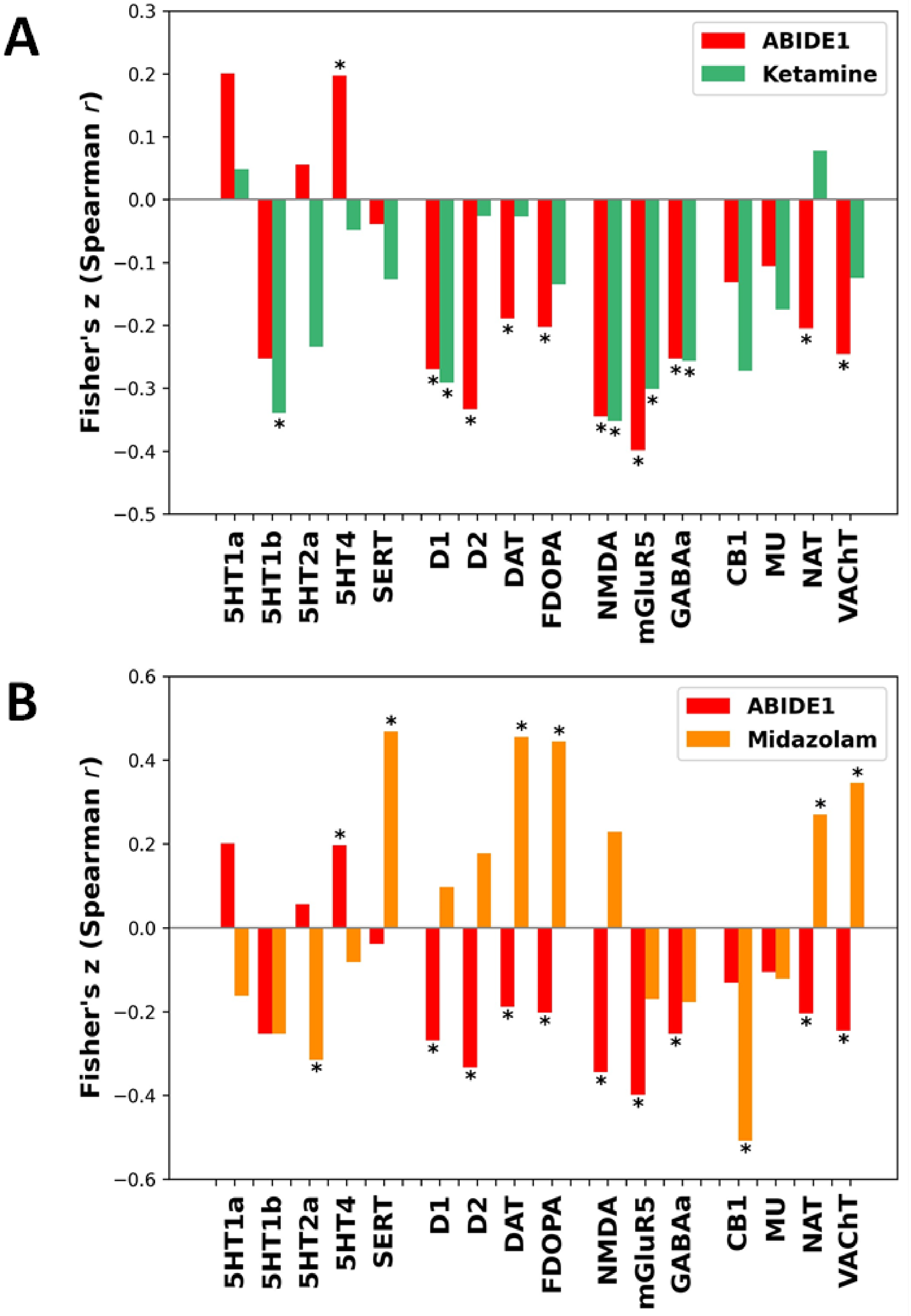

Supplementary Fig. S3. Neurotransmitter co-localizations with LCOR changes induced by ketamine and midazolam (n = 27) compared to the co-localization profile of individuals with autism (n = 405) compared to typically developed controls (TD; n = 473) in ABIDE1. (A) Two-sided spatial co-localizations (Spearman correlations) of neurotransmitter distributions with local synchronization (LCOR) alterations induced by NMDA antagonist ketamine (versus placebo) compared to the co-localization profile in autism (versus TD) in ABIDE1. (B) Two-sided spatial co-localizations (Spearman correlations) of neurotransmitter distributions with LCOR alterations induced by GABAa potentiator midazolam (versus placebo) compared to the co-localization profile in autism (versus TD) in ABIDE1. The asterisk (*) represents significant co-localizations ($p < .05$). df = 117. 5HT = 5-hydroxytryptamine (serotonin receptor); SERT = serotonin transporter; D1/D2 = dopamine receptor; DAT = dopamine transporter; FDOPA = fluorodopa; NMDA = N-Methyl-D-Aspartat receptor; mGluR5 = metabotropic glutamate receptor; GABAa = γ-aminobutyric acid type A receptor; CB1 = cannabinoid receptor; MU = μ-opioid receptor; NAT = noradrenaline transporter; VAChT = vesicular acetylcholine transporter. Source data are provided as a Source Data file. Voxel-wise NIfTI files are available from the public repository (reference).

Supplementary Tables S11-14. LCOR alterations induced by ketamine and midazolam administration

We computed voxel-wise whole-brain comparisons of LCOR induced by ketamine and midazolam administration compared to placebo within the CONN toolbox[60].

Ketamine induced LCOR reductions in pre- and postcentral gyri, frontal cortices, precuneus, cingulate, insular and opercular cortices, whereas LCOR was increased particularly in the cerebellum and temporal regions. Midazolam induced LCOR reductions in the left lateral occipital cortex and increases within the left hemispheric supplementary motor cortex, precentral gyrus, putamen, insular cortex and middle temporal gyrus as well as anterior cingulate cortex.

contrast	cluster (x, y, z)	size	peaks	TFCE	peak *p-FWE*	peak *p-FDR*
**KET>PLC**	+22 -14 -36	847	20	925.07	.0010	.0012
	+24 -42 -48	783	17	875.15	.0030	.0012
	+38 -52 -30	90	4	730.68	.0110	.0012
	-34 -82 +12	106	1	723.52	.0110	.0012
	-40 -52 -42	97	1	719.73	.0120	.0012
	+28 -46 -16	95	4	712.09	.0120	.0012
	+02 -82 -42	107	2	661.03	.0230	.0017
	+52 +00 +14	28	1	640.31	.0320	.0021
	-10 -72 -48	20	1	625.02	.0350	.0021
	-24 -64 -50	46	3	619.83	.0390	.0021
**KET<PLC**	-06 -28 +62	2302	29	-1596.81	< .0001	< .0001
	-36 +40 +02	449	11	-989.32	.0020	.0014
	+50 +26 -14	368	9	-941.97	.0020	.0014
	+08 +32 +22	339	9	-904.17	.0030	.0015
	-50 +10 -08	99	4	-631.31	.0380	.0025
	-34 +12 -04	67	2	-630.59	.0390	.0025
	+44 +44 -18	13	1	-611.86	.0430	.0026
	+34 +58 -18	29	1	-595.40	.0470	.0028

Supplementary Table S11. Main effect of ketamine on LCOR compared to placebo condition (n = 27). List of significant TFCE-corrected cluster for positive (ketamine>placebo) and negative (ketamine<placebo) contrast (*t*-tests); highlighted cluster coordinates indicate the voxel with the highest T-value within a cluster; KET = ketamine; PLC = placebo; TFCE = threshold free cluster enhancement; FDR = false discovery rate; FWE = family-wise error; LCOR = local synchronization; TFCE-threshold: $p < 0.05$. Voxel-wise NIfTI files are available from the public repository (reference).

Contrast: KET>PLC
286 voxels (13%) covering 36% of atlas.Cereb9 r (Cerebelum 9 Right)
167 voxels (8%) covering 57% of atlas.aTFusC r (Temporal Fusiform Cortex, anterior division Right)
163 voxels (7%) covering 7% of atlas.Cereb8 r (Cerebelum 8 Right)
128 voxels (6%) covering 5% of atlas.TP r (Temporal Pole Right)
111 voxels (5%) covering 17% of atlas.aPaHC r (Parahippocampal Gyrus, anterior division Right)
102 voxels (5%) covering 12% of atlas.Cereb9 l (Cerebelum 9 Left)
94 voxels (4%) covering 4% of atlas.Cereb1 l (Cerebelum Crus1 Left)
92 voxels (4%) covering 2% of atlas.sLOC l (Lateral Occipital Cortex, superior division Left)
77 voxels (3%) covering 9% of atlas.TOFusC r (Temporal Occipital Fusiform Cortex Right)
68 voxels (3%) covering 2% of atlas.Brain-Stem
67 voxels (3%) covering 4% of atlas.Cereb8 l (Cerebelum 8 Left)
51 voxels (2%) covering 16% of atlas.aITG r (Inferior Temporal Gyrus, anterior division Right)
48 voxels (2%) covering 2% of atlas.Cereb2 r (Cerebelum Crus2 Right)
44 voxels (2%) covering 3% of atlas.Cereb6 r (Cerebelum 6 Right)
39 voxels (2%) covering 5% of atlas.pTFusC r (Temporal Fusiform Cortex, posterior division Right)
28 voxels (1%) covering 1% of atlas.Cereb1 r (Cerebelum Crus1 Right)
27 voxels (1%) covering 17% of atlas.Cereb10 r (Cerebelum 10 Right)
16 voxels (1%) covering 1% of atlas.LG r (Lingual Gyrus Right)
12 voxels (1%) covering 2% of atlas.Cereb7 r (Cerebelum 7b Right)
9 voxels (0%) covering 0% of atlas.PreCG r (Precentral Gyrus Right)
7 voxels (0%) covering 1% of atlas.Cereb7 l (Cerebelum 7b Left)
6 voxels (0%) covering 2% of atlas.Amygdala r
3 voxels (0%) covering 0% of atlas.Hippocampus r
2 voxels (0%) covering 0% of atlas.Cereb2 l (Cerebelum Crus2 Left)
572 voxels (26%) covering 0% of atlas.not-labeled
Contrast: KET<PLC
839 voxels (23%) covering 20% of atlas.PreCG r (Precentral Gyrus Right)
491 voxels (13%) covering 11% of atlas.PreCG l (Precentral Gyrus Left)
396 voxels (11%) covering 6% of atlas.FP l (Frontal Pole Left)
331 voxels (9%) covering 10% of atlas.PostCG r (Postcentral Gyrus Right)
308 voxels (8%) covering 12% of atlas.AC (Cingulate Gyrus, anterior division)
183 voxels (5%) covering 5% of atlas.PostCG l (Postcentral Gyrus Left)
150 voxels (4%) covering 10% of atlas.FOrb r (Frontal Orbital Cortex Right)
88 voxels (2%) covering 7% of atlas.IC l (Insular Cortex Left)
83 voxels (2%) covering 1% of atlas.FP r (Frontal Pole Right)
50 voxels (1%) covering 2% of atlas.TP l (Temporal Pole Left)
33 voxels (1%) covering 1% of atlas.TP r (Temporal Pole Right)
25 voxels (1%) covering 2% of atlas.PaCiG r (Paracingulate Gyrus Right)
18 voxels (0%) covering 0% of atlas.Precuneous (Precuneous Cortex)
16 voxels (0%) covering 3% of atlas.IFG tri r (Inferior Frontal Gyrus, pars triangularis Right)
16 voxels (0%) covering 2% of atlas.IFG oper r (Inferior Frontal Gyrus, pars opercularis Right)
12 voxels (0%) covering 1% of atlas.PC (Cingulate Gyrus, posterior division)
4 voxels (0%) covering 0% of atlas.CO l (Central Opercular Cortex Left)
3 voxels (0%) covering 0% of atlas.MidFG l (Middle Frontal Gyrus Left)
3 voxels (0%) covering 1% of atlas.FO l (Frontal Operculum Cortex Left)

2 voxels (0%) covering 0% of atlas.IC r (Insular Cortex Right)
2 voxels (0%) covering 0% of atlas.SMA r (Juxtapositional Lobule Cortex -formerly Supplementary Motor Cortex- Right)
1 voxels (0%) covering 0% of atlas.IFG tri l (Inferior Frontal Gyrus, pars triangularis Left)
1 voxels (0%) covering 0% of atlas.PaCiG l (Paracingulate Gyrus Left)
1 voxels (0%) covering 0% of atlas.FO r (Frontal Operculum Cortex Right)
610 voxels (17%) covering 0% of atlas.not-labeled

Supplementary Table S12. Regions with significant LCOR alterations induced by ketamine (all clusters combined, n = 27). List of all significant voxels and atlas regions with altered LCOR induced by ketamine compared to placebo for the positive (KET>PLC) and negative contrast (KET<PLC); KET = ketamine; PLC = placebo; LCOR = local synchronization. Voxel-wise NIfTI files are available from the public repository (reference).

contrast	cluster (x, y, z)	size	peaks	TFCE	peak p-FWE	peak p-FDR
**MDZ>PLC**	-08 -14 +44	83	3	685.91	.0330	.0184
	-32 -02 +02	20	1	678.34	.0330	.0184
	-46 -20 -14	37	1	657.11	.0430	.0184
**MDZ<PLC**	-34 -74 +34	120	1	-821.65	.0060	.0188

Supplementary Table S13. Main effect of midazolam on LCOR compared to placebo condition (n = 27). List of significant TFCE-corrected cluster for positive (midazolam>placebo) and negative (midazolam<placebo) contrast (*t*-tests); highlighted cluster coordinates indicate the voxel with the highest T-value within a cluster; MDZ = midazolam; PLC = placebo; TFCE = threshold free cluster enhancement; FDR = false discovery rate; FWE = family-wise error; LCOR = local synchronization; TFCE-threshold: $p < 0.05$. Voxel-wise NIfTI files are available from the public repository (reference).

Contrast: MDZ>PLC

29 voxels (21%) covering 5% of atlas.SMA L (Supplementary Motor Cortex- Left)
20 voxels (14%) covering 0% of atlas.PreCG l (Precentral Gyrus Left)
7 voxels (5%) covering 1% of atlas.Putamen l
4 voxels (3%) covering 0% of atlas.IC l (Insular Cortex Left)
4 voxels (3%) covering 0% of atlas.pMTG l (Middle Temporal Gyrus, posterior division Left)
3 voxels (2%) covering 0% of atlas.AC (Cingulate Gyrus, anterior division)
1 voxels (1%) covering 0% of atlas.PP l (Planum Polare Left)
72 voxels (51%) covering 0% of atlas.not-labeled

Contrast: MDZ<PLC

120 voxels (100%) covering 2% of atlas.sLOC l (Lateral Occipital Cortex, superior division Left)

Supplementary Table S14. Regions with significant LCOR alterations induced by midazolam (all clusters combined, n = 27). List of all significant voxels and atlas regions with altered LCOR induced by midazolam compared to placebo for the positive (MDZ>PLC) and negative contrast (MDZ<PLC); MDZ = midazolam; PLC = placebo; LCOR = local synchronization. Voxel-wise NIfTI files are available from the public repository (reference).

Supplementary Figures S4-5. Visualization of ketamine and midazolam induced LCOR changes

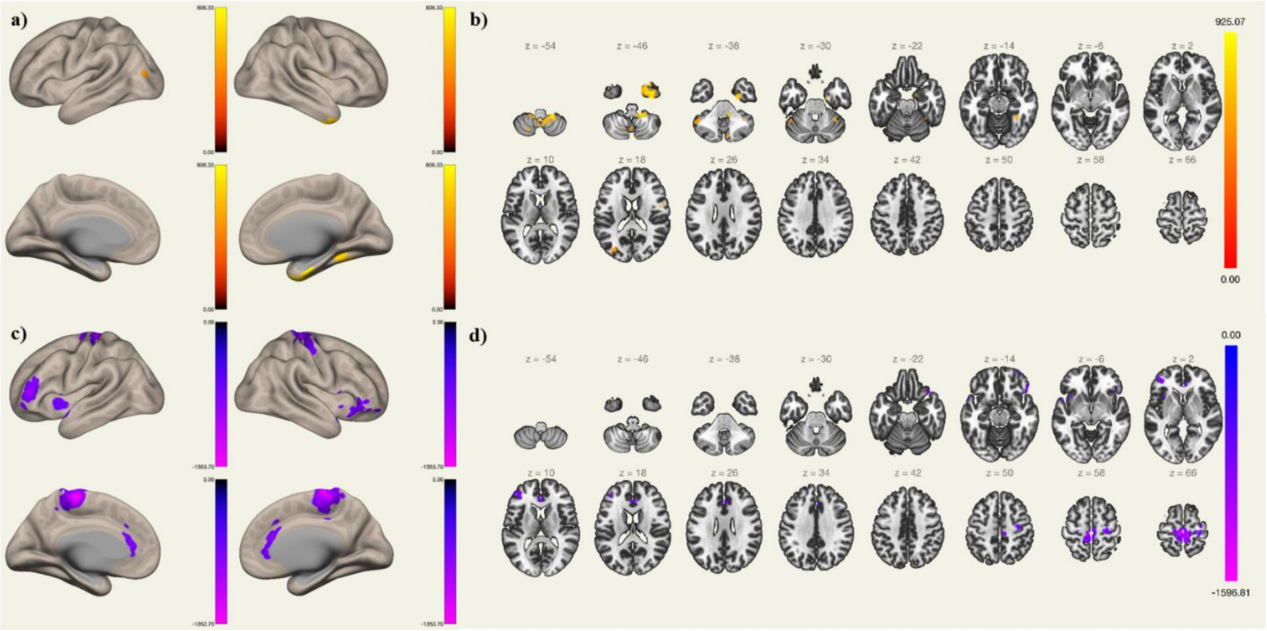

Supplementary Fig. S4. Significant voxel-wise LCOR changes (two-sided t-tests) after ketamine administration compared to placebo condition (n = 27). Increased LCOR induced by ketamine compared to placebo (red-yellow), a) sagittal view and b) axial view. Decreased LCOR after ketamine administration compared to placebo (blue-purple), c) sagittal view and d) axial view. Voxel-wise family-wise error threshold of $p < 0.05$ combined with an exact permutation-based cluster threshold (threshold free cluster enhancement (TFCE), 1000 permutations, $p < 0.05$). Voxel-wise NIfTI files are available from the public repository (reference).

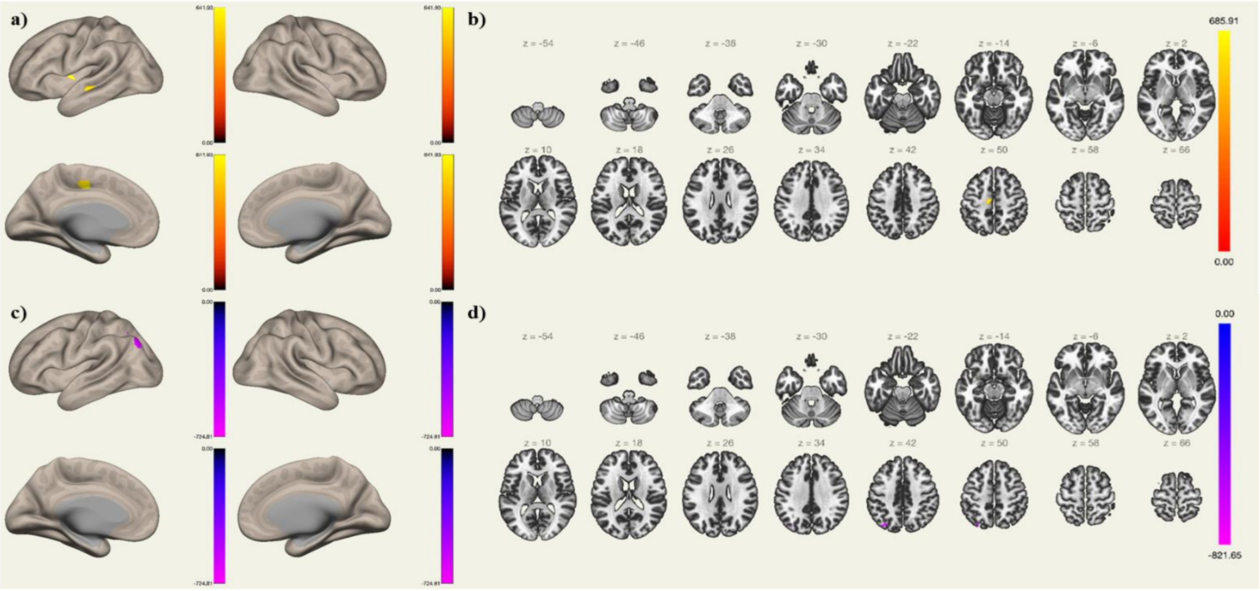

Supplementary Fig. S5. Significant voxel-wise LCOR changes (two-sided t-tests) after midazolam administration compared to placebo condition (n = 27). Increased LCOR induced by midazolam compared to placebo (red-yellow), a) sagittal view and b) axial view. Decreased LCOR after midazolam administration compared to placebo (blue-purple), c) sagittal view and d) axial view. Voxel-wise family-wise error threshold of $p < 0.05$ combined with an exact permutation-based cluster threshold (threshold free cluster enhancement (TFCE), 1000 permutations, $p < 0.05$). Voxel-wise NIfTI files are available from the public repository (reference).

Supplementary Table S15. Similarity of autism-related and medication effects

To assess the similarity between the brain pattern observed in autism and the LCOR alterations induced by ketamine and midazolam, we conducted a correlation analysis within the JuSpace toolbox[65] using LCOR T-maps generated by the CONN toolbox[60]. Our findings reveal a significant positive correlation between ketamine-induced and autism-related global brain patterns, indicating that ketamine elicits autism-like local activity patterns. In contrast, midazolam did not show a similar correlation.

Furthermore, while the whole-brain LCOR T-maps for ketamine and midazolam were not significantly correlated with each other, the LCOR patterns observed in the ABIDE1 and ABIDE2 datasets demonstrated a strong correlation.

	**Pearson *r***	***p***
ABIDE I vs. KET	.381	.003**
ABIDE I vs. MDZ	.018	.903
ABIDE II vs. KET	.292	.036*
ABIDE II vs. MDZ	.205	.155
ABIDE I vs. ABIDE II	.597	< .001***
KET vs. MDZ	.075	.637

Supplementary Table S15. Correlation (two-sided) between autism-specific and pharmaco-induced T-maps. ABIDE = Autism Brain Imaging Data Exchange; KET = ketamine; MDZ = midazolam; TD = typically developed controls; autism contrast (autism>TD) vs. ketamine contrast (KET>PLC) vs. midazolam contrast (MDZ>PLC). Source data are provided as a Source Data file. Df = 117. Autism data (n = 405, n = 396); TD data (n = 473, n = 474); pharma data (n = 27). Voxel-wise NIfTI files are available from the public repository (reference).

Supplementary Table S16. Correlation of the LCOR-neurotransmitter co-localizations with autism symptom domains in ABIDE1 and ABIDE2

We extracted individual Fisher's z scores for all subjects with autism from ABIDE1 and ABIDE2 separately and computed Pearson correlation analyses with the autism diagnostic observation schedule (ADOS) subscales[67]. For the consistent significant LCOR-neurotransmitter co-localizations observed across both ABIDE datasets, we did not find any significant association with clinical symptom domains in ABIDE1. The co-localization with GABAa and VAChT was significantly associated with stereotyped behavior and restricted interests (SBRI) in ABIDE2.

		Communication		Social interaction		SBRI	
		r	*p*	*r*	*p*	*r*	*p*
5HT1a	ABIDE1	-.013	.821	-.014	.806	-.016	.806
	ABIDE2	.009	.889	-.026	.692	.016	.809
5HT1b	ABIDE1	.127	.029*	-.049	.394	.009	.893
	ABIDE2	-.055	.401	.036	.578	-.046	.483
5HT2a	ABIDE1	.047	.421	-.052	.363	.026	.685
	ABIDE2	-.062	.342	-.047	.470	-.088	.176
5HT4	ABIDE1	-.057	.324	-.045	.440	-.025	.701
	ABIDE2	-.147	.024*	-.053	.411	.044	.495
SERT	ABIDE1	-.116	.046*	.003	.964	-.109	.087
	ABIDE2	-.110	.089	-.038	.559	.034	.601
D1	ABIDE1	-.047	.417	.010	.857	-.016	.797
	ABIDE2	-.095	.146	.046	.481	.113	.083
D2	ABIDE1	-.016	.777	-.009	.878	-.046	.476
	ABIDE2	-.078	.233	.007	.909	-.015	.820
DAT	ABIDE1	-.055	.346	.046	.427	-.061	.341
	ABIDE2	-.071	.273	.019	.768	.105	.105
FDOPA	ABIDE1	-.060	.303	.061	.286	-.071	.264
	ABIDE2	-.042	.522	.024	.712	.065	.317
NMDA	ABIDE1	-.032	.580	.011	.849	-.112	.080
	ABIDE2	-.092	.155	-.023	.730	-.039	.548
mGluR5	ABIDE1	.035	.552	-.027	.644	.021	.746
	ABIDE2	-.022	.733	.050	.445	-.065	.321
GABAa	ABIDE1	.077	.185	-.044	.445	.028	.663
	ABIDE2	-.014	.833	-.004	.948	-.133	.040*
CB1	ABIDE1	.049	.401	-.043	.456	.073	.253
	ABIDE2	-.031	.630	.063	.330	< .001	.996
MU	ABIDE1	< .001	.998	-.005	.933	.033	.608
	ABIDE2	-.014	.836	.097	.136	.171	.008**
NAT	ABIDE1	.075	.199	.094	.101	-.057	.369
	ABIDE2	.028	.668	-.049	.454	-.044	.502
VAChT	ABIDE1	-.034	.555	.076	.189	-.032	.613
	ABIDE2	-.027	.679	.040	.536	.141	.030*

Supplementary Table S16. Pearson correlations between the strength of LCOR-neurotransmitter co-localizations in autism and the ADOS subscales (ABIDE1: n = 300; ABIDE2: n = 238). ADOS = autism diagnostic observation schedule; LCOR = local synchronization; SBRI = stereotyped behavior and restricted interests; 5HT = 5-hydroxytryptamine (serotonin receptor); SERT = serotonin transporter; D1/D2 = dopamine receptor; DAT = dopamine transporter; FDOPA = fluorodopa; NMDA = N-Methyl-D-Aspartat receptor; mGluR5 = metabotropic glutamate receptor; GABAa = γ-aminobutyric acid type A receptor; CB1 = cannabinoid receptor; MU = μ-opioid receptor; NAT = noradrenaline transporter; VAChT = vesicular acetylcholine transporter; CBF = cerebral blood flow; * = $p < .05$; ** = $p < .01$; *** = $p < .001$. Source data are provided as a Source Data file.

Supplementary Fig. S6. Correlation of the LCOR-neurotransmitter co-localizations in autism with clinical symptom domains

There was no significant association between the consistent LCOR-neurotransmitter co-localizations and clinical symptom severity as measures with the ADOS score in the ABIDE1 dataset. A significant but weak negative association was observed in ABIDE2 between SBRI and the strength of LCOR-GABAa co-localizations (r = -.133, p = .040) and a positive with the strength of LCOR-VAChT co-localizations (r = .141, p = .030). Both findings did not survive a correction for multiple comparisons.

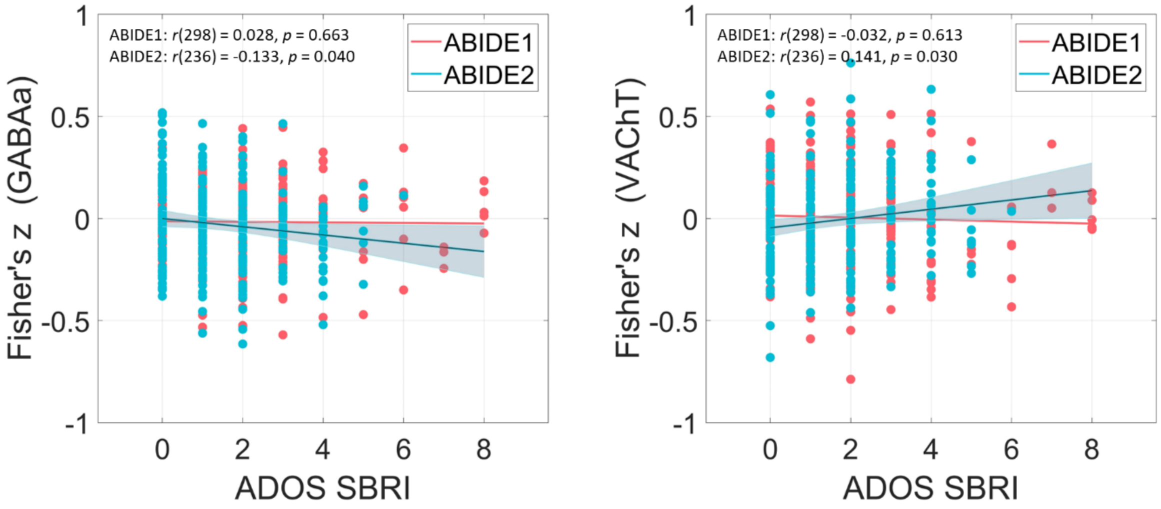

Supplementary Fig. S6. Association of the co-localizations between LCOR and GABAa and VAChT distributions with stereotyped behavior and restricted interests (SBRI). Scatterplot showing values of the SBRI subscale of the Autism Diagnostic Observation Schedule (ADOS) relative to the strength of the spatial co-localization (individual Fisher's z-scores) between local synchronization (LCOR) and GABAa as well as VAChT distributions in subjects with autism (ABIDE1: n = 300; ABIDE2: n = 238). GABAa = γ-aminobutyric acid type A receptor; VAChT = vesicular acetylcholine transporter. Trend lines represent the least-squares linear fit; shaded area denotes the 95 % confidence interval. Statistical analysis was performed using two-sided

Pearson correlations (before multiple comparison correction). Source data are provided as a Source Data file.

Materials and Methods

Supplementary Fig. S7. Exclusion process

We excluded subjects with intellectual disability (IQ ≤ 70), subjects with missing information and subjects with excessive head motion during image acquisition (translation > 3 mm or rotation > 3°). For ABIDE2, another 3 subjects were excluded due to preprocessing failure of functional imaging data.

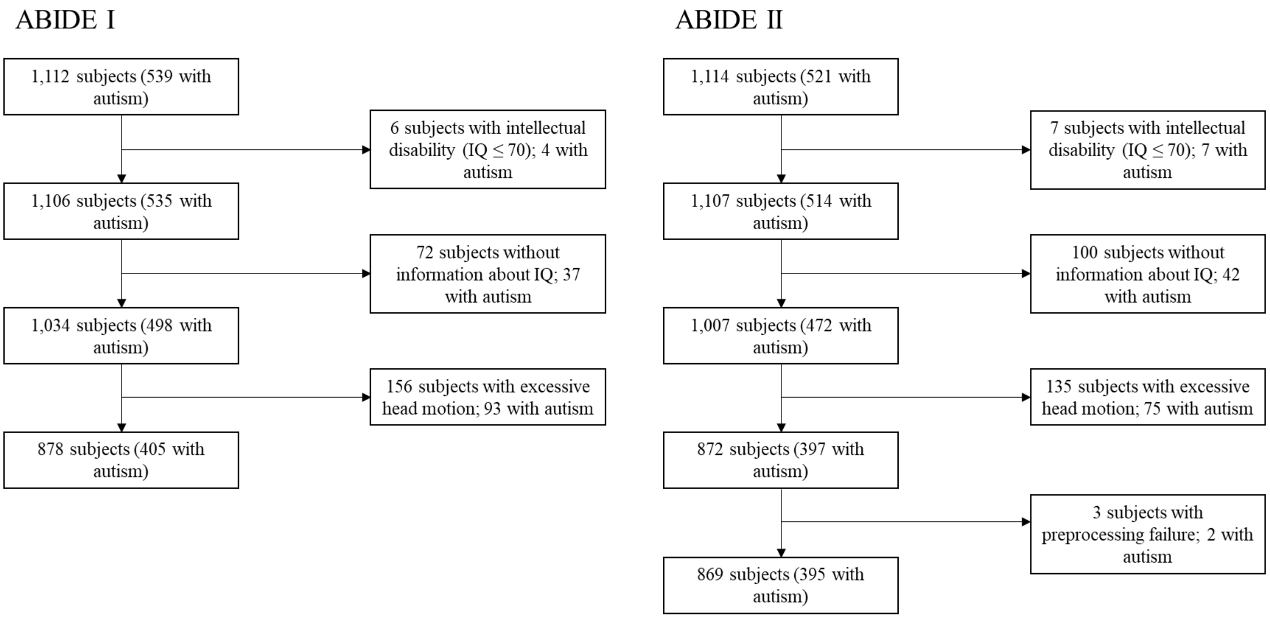

Supplementary Fig. S7. Overview of the exclusion process for the Autism Brain Imaging Data Exchange (ABIDE) 1 and ABIDE2 datasets, IQ = intelligence quotient.

Supplementary Tables S17-18. MRI data acquisition

Supplementary Table S17. Detailed description of MRI acquisition parameters of the respective sites included in ABIDE I.

**Scanner characteristics**												
**Site**	Caltech	KKI	Leuven	MaxMun	NYU	Olin	Pitt	SDSU	Trinity	UCLA	USM	Yale
**Manufacturer, type**	Siemens, MAGNETOM TIM Trio	Philips, Achieva	Philips, NA	Siemens, MAGNETOM Verio	Siemens, MAGNETOM Allegra	Siemens, MAGNETOM Allegra	Siemens, MAGNETOM Allegra	GE, MR750	Philips, Achieva	Siemens, MAGNETOM TIM Trio	Siemens, MAGNETOM TIM Trio	Siemens, MAGNETOM TIM Trio
**Field strength**	3T	3T	3T	3T	3T	3T	3T	3T	3T	3T	3T	3T
**Headcoil**	NA	NA	NA	NA	NA	NA	NA	8-ch	8-ch	NA	NA	NA
**T1-weighted image acquisition**												
**TR/TE/TI (ms)/flip angle (°)**	1590/ 2.73/800/10	min/ min/1000/8	min/ 4.6/900/8	1800/ 3.06/900/9	2530/ 3.25/1100/7	2500/ 2.74/900/8	2100/ 3.93/1000/7	NA/ min /600/8	8.5 3.9/3000/ 8	2300/ 2.84/853/9	2300/ 2.91/900/9	1230/ 1.73/624/9
**No. of slices**	176	200	182	160	128	176	176	176	160	160	160	176
**Voxel size (mm²)**	1.0 x 1.0	1.0 x 1.0	1.0 x 1.0	1.0 x 1.0	1.3 x 1.0	1.0 x 1.0	1.1 x 1.1	1.0 x 1.0	1.0 x 1.0	1.0 x 1.0	1.0 x 1.0	1.0 x 1.0
**Slice thickness (mm)**	1.0	1.0	1.2	1.0	1.3	1.0	1.1	1.0	1.0	1.2	1.2	1.0
**EPI BOLD image acquisition**												
**Instruction**	Eyes closed	Fixation	Fixation	Fixation or eyes closed	Fixation	Fixation	Eyes closed	Fixation	Eyes closed	Fixation	Eyes open	Eyes open
**TR/TE (ms)/flip angle (°)**	2000/ 30/75	2500/ 30/75	1667/ 33/90	3000/ 30/80	2000/ 15/90	1500/ 27/60	1500/ 25/70	2000/ 30/90	2000/ 28/90	3000/ 28/90	2000/ 28/90	2000/ 25/60
**No. of volumes**	150	156	250	120/200	180	210	200	180	150	120	240	200
**No. of slices**	34	47	32	40	33	29	29	41	38	34	40	34
**Voxel size (mm²)**	3.5 × 3.5	3.0 × 3.0	3.6 × 3.6	3.0 × 3.0	3.0 × 3.0	3.4 × 3.4	3.1 × 3.1	3.4 × 3.4	3.0 × 3.0	3.0 × 3.0	3.4 × 3.4	3.4 × 3.4
**Slice thickness (mm)**	3.5	3.0	4.0	3.0	4.0	4.0	4.0	3.4	3.5	4.0	3.0	4.0

Supplementary Table S18. Detailed description of MRI acquisition parameters of the respective sites included in ABIDE II.

**Scanner characteristics**														
**Site**	ETH	GU	IU	KKI	KUL	NYU1	NYU2	OHSU	ONRC	SDSU	TCD	UCLA	UCD	USM
**Manufacturer, type**	Philips, Achieva	Siemens, MAGNETOM TIM Trio	Siemens, MAGNETOM TIM Trio	Philips, Achieva	Philips, NA	Siemens, MAGNETOM Allegra	Siemens, MAGNETOM Allegra	Siemens, MAGNETOM TIM Trio	Siemens, MAGNETOM Skyra	GE, MR750	Philips, Achieva	Siemens, MAGNETOM TIM Trio	Siemens, MAGNETOM TIM Trio	Siemens, MAGNETOM TIM Trio
**Field strength**	3T	3T	3T	3T	3T	3T	3T	3T	3T	3T	3T	3T	3T	3T
**Headcoil**	32-ch	12-ch	32-ch	8/32-ch	32-ch	NA	NA	12-ch	NA	8-ch	8-ch	12-ch	8-ch	12-ch
**T1-weighted image acquisition**														
**TR/TE/TI (ms)/flip angle (°)**	8.4/ min/1150 /8	2530/ 3.5/1100/ 7	2400/ 2.3/1000/ 8	min/ min/1000 /8	min 4.6/900 /8	2530/ 3.25/1100 /7	2530/ 3.25/1100 /7	2300/ 3.58/900/ 10	2200/ 2.88/794/ 13	NA/ min/600 /8	8.4/ 3.9/1150 /8	2300/ 2.86/853/ 9	2000/ 3.16/1050 /8	900/ 2.91/900/ 9
**No. of slices**	180	176	256	200	182	128	128	160	208	176	190	160	192	160
**Voxel size (mm^2)**	0.9 x 0.9	1.0 x 1.0	0.7 x 0.7	1.0 x 1.0	1.2 x 1.0	1.0 x 1.2	1.3 x 1.0	1.0 x 1.0	0.8 x 0.8	1.0 x 1.0	0.9 x 0.9	1.0 x 1.0	1.0 x 1.0	1.0 x 1.0
**Slice thickness (mm)**	0.9	1.0	0.7	1.0	1.2	1.3	1.3	1.1	0.8	1.0	0.9	1.2	1.0	1.2
**EPI BOLD image acquisition**														
**Instruction**	Fixation	Eyes open	Eyes open	Fixation	Fixation	Fixation	Fixation	Fixation	Fixation	Fixation	Fixation	Fixation	Eyes open	Eyes open
**TR/TE (ms)/flip angle (°)**	2000/ 25/90	2000/ 30/90	813/ 28/60	2500/ 30/75	2500/ 30/90	2000/ 15/90	2000/ 30/82	2000/ 30/90	475/ 30/60	2000/ 30/90	2000/ 27/90	3000/ 28/90	2000/ 24/90	2000/ 28/90
**No. of volumes**	210	154	433	156	162	180	180	120	947	180	210	120	460	240
**No. of slices**	40	43	42	47	45	33	34	36	48	41	37	34	36	40
**Voxel size (mm^2)**	3.0 × 3.1	3.0 × 3.0	3.4 × 3.4	3.0 × 3.0	2.5 × 2.5	3.0 × 3.0	3.0 × 3.0	3.8 × 3.8	3.0 × 3.0	3.4 × 3.4	3.0 × 3.0	3.0 × 3.0	3.5 × 3.5	3.4 × 3.4
**Slice thickness (mm)**	3.0	2.5	3.4	3.0	2.7	4.0	3.0	3.8	3.0	3.4	3.2	4.0	4.0	3.0

THE UNIVERSITY OF AUCKLAND
NEW ZEALAND
Te Whare Wānanga o Tāmaki Makaurau

Study Title: A simultaneous EEG/fMRI crossover study of ketamine, placebo and midazolam

Short Title: EFKAM

Principal Investigator:

Dr Suresh Muthukumaraswamy
Senior Research Fellow
School of Pharmacy
Auckland University

Co-Investigators:

Ms Anna Forsyth
PhD Candidate
School of Pharmacy
Auckland University

Dr Gemma Malpas
Fellow in Neuroanaesthesia
Auckland City Hospital
Auckland District Health Board

Dr Liz Maxwell
Fellow in Neuroanaesthesia
Auckland City Hospital
Auckland District Health Board

Dr Jörg Hipp
F. Hoffmann-La Roche Ltd
Grenzacherstrasse 124
4070 Basel
Switzerland

Dr Juergen Dukart
F. Hoffmann-La Roche Ltd
Grenzacherstrasse 124
4070 Basel
Switzerland

Funders:

The Royal Society of New Zealand
F. Hoffmann-La Roche Ltd
The University of Auckland

Study Sponsor:

The University of Auckland

TABLE OF CONTENTS

1. KEY STUDY CONTACTS 4
2. ABBREVIATIONS 5
3. SYNOPSIS 6
4. BACKGROUND AND RATIONALE 7
5. AIMS 8
6. LIST OF MEASURES 8
7. STUDY DESIGN 9
8. PARTICIPANT IDENTIFICATION 10
8.1. Study Participants 10
8.2. Inclusion Criteria 10
8.3. Exclusion Criteria 10
8.4. Pilot participants 11
8.5. Loss of participants 11
8.6. Payment of participants 11
9. STUDY PROCEDURES 11
9.1. Recruitment 11
9.2. Informed Consent 11
9.3. Randomisation and Blinding 12
9.4. Discontinuation/Withdrawal of Participants from Study Treatment 12
9.5. Definition of End of Study 12
9.6. Schedule of Procedures 13
9.7. Summary of Procedures 13
10. INVESTIGATIONAL MEDICINAL PRODUCTS (IMP) 15
10.1. Storage of Ketamine and Midazolam 15
10.2. Accountability of the Study Treatments 16
11. SAFETY 16
11.1. Definitions 16
11.2. Causality 16
11.3. Procedures for Recording Adverse Events 17
11.4. Reporting Procedures for Serious Adverse Events 17
11.5. Safety Monitoring Committee 17
11.6 Safety Protocols 17

12. DATA ANALYSIS AND STATISTICS ... 18
12.1. Power calculations ... 18
12.2. Analytical Methods ... 18
13. DATA MANAGEMENT ... 18
13.1. Access to Data ... 18
13.2. Data Recording and Record Keeping ... 18
13.3 Electronic Data Storage ... 18
14. STUDY STEERING COMMITTEE ... 19
15. ETHICAL AND REGULATORY CONSIDERATIONS ... 19
15.1. Declaration of Helsinki ... 19
15.2. ICH Guidelines for Good Clinical Practice ... 19
15.3. Approvals ... 19
15.4. Reporting ... 19
15.5. Participant Confidentiality ... 19
15.6. Additional Ethical Considerations ... 19
16. FUNDING ... 21
17. REFERENCES ... 21

THE UNIVERSITY OF AUCKLAND
NEW ZEALAND
Te Whare Wānanga o Tāmaki Makaurau

1. KEY STUDY CONTACTS

**Principal Investigator**	Dr Suresh Muthukumaraswamy<br>Senior Research Fellow<br>Schools of Pharmacy and Psychology<br>University of Auckland<br>Private Bag 92019<br>Auckland 1142<br>New Zealand<br>Phone: +64 9 373 7599 ext 85398<br>Email: sd.muthu@auckland.ac.nz
**Research Facility**	Centre for Advanced Magnetic Resonance Imaging (CAMRI)<br>Basement Level, Building 505<br>Faculty of Medical and Health Sciences<br>85 Park Road<br>Grafton<br>Auckland New Zealand<br>Phone: 09 306 5966<br>Email: mri@auckland.ac.nz
**Institutional Contact**	Dr Jeff Harrison<br>Acting Head of School of Pharmacy<br>The University of Auckland,<br>Private Bag 92019, Auckland.<br>Tel: 373-7599 ext 85262<br>Email: j.harrison@auckland.ac.nz

2. ABBREVIATIONS

5D-ASC	5 Dimensional Altered States of Consciousness
AE	Adverse event
ASL	Arterial Spin Labelling
CAMRI	Centre for Advanced Magnetic Resonance Imaging
CF	Consent Form
CRF	Case Report Form
EEG	Electroencephalogram
FMRI	Functional Magnetic Resonance Imaging
GABA	Gamma-Aminobutyric Acid
GCP	Good Clinical Practice
GP	General Practitioner
HDEC	Health and Disability Ethics Committee
IV	Intravenous
MID	Monetary Incentive Delay
NMDA	N-Methyl-D-Aspartate
PI	Principal Investigator
PIS	Participant Information Sheet
SAE	Serious Adverse Event
SAR	Serious Adverse Reaction
SOP	Standard Operating Procedure
SUSAR	Suspected Unexpected Serious Adverse Reactions
TMF	Trial Master File

3. SYNOPSIS

Study Title	A simultaneous EEG/fMRI crossover study of ketamine, placebo and midazolam
Study Design	Randomised, single-blind, placebo-controlled, crossover
Study Participants	Healthy male volunteers
Planned Sample Size	n = 30
Treatment duration	3 days, single dose
Follow up duration	1-2 hours each day
Planned Study Period	1st March 2016 – 1st February 2017
Primary Measures	EEG<br>fMRI<br>ASL
Investigational Medicinal Products	Racemic ketamine (Ketalar®)<br>Midazolam
Dose and Route of Administration[1]	Ketamine IV. – bolus dose up to 0.5mg/kg then infusion at up to 0.6 mg/kg/hr.<br>Midazolam IV – bolus dose up to 0.1mg/kg then infusion at up to 0.1 mg/kg/hr.

[1] These doses are a safe upper window of the doses to be used in the experiment. Exact doses will be determined during pilot testing.

4. BACKGROUND AND RATIONALE

EEG and fMRI are two of the principal techniques currently used to measure human brain function. These two techniques provide access to different aspects of neuronal activity. EEG provides direct access to electrophysiological population-level activity at a coarse spatial resolution, while fMRI and ASL measure blood oxygenation and blood flow reflecting metabolic costs of neuronal population activity at a high spatial resolution. In principle, the simultaneous acquisition of the two types of measures, i.e. quantifying electrophysiological signals with their associated metabolic costs, should provide more insights than either technique alone. The combined acquisition of EEG and fMRI is technically possible, but knowledge on the advantages of using the synergistic combination of the two approaches remains scarce. In particular, it is unknown how EEG activity and connectivity across different frequencies translate into metabolic demands. Understanding this relationship is critical to applying these techniques to measurements made in the context of drug and disease states.

While there has been a long history of studying drug-induced modifications of the EEG [1], there is a much smaller literature on the use of fMRI and ASL in quantifying pharmacologically-induced modifications of brain responses at a circuitry level. While fMRI/ASL have high spatial resolution, their generative mechanisms fundamentally rely on blood flow and vascular responses. Because drugs can potentially modify both the neuronal and vascular properties of the brain, uncertainty can exist as to which one of these factos may be contributing to the responses [2]. Generally speaking, for neuroscientific applications it is desirable to isolate neuronal responses, whereas vascular responses are considered a confound. EEG, on the otherhand is not affected by vascular responses. Therefore the simultaneous acquisition of EEG/fMRI and joint data analysis presents an opportunity to overcome the difficulties of vascular confounds and more precisely understand the electrophysiological signatures underlying fMRI signals in specific therapeutic contexts. Improved knowledge of the neuronal basis of pharmacological modifications of fMRI could help to disentangle equivocal findings in each modality alone (see below). Improved localization and understanding of pharmaco-induced neuro-metabolic interactions on the brain circuitry level could also facilitate the choice of indications to be targetted in the drug development process.

In this study we aim to explore the synergistic potential of simultaneously recorded EEG and fMRI in a single blinded randomized cross-over study with placebo, ketamine, and midazolam. This work will provide a proof of concept as to how to employ the combination of EEG and fMRI in a drug study and will allow exploration of the potential benefits of this combination in the analysis of both resting-state and task-based fMRI/EEG data.

<u>Choice of medications</u>

In this study we will modify brain activity using ketamine and midazolam. Both drugs have relatively rapid effects on brain activity when delivered by intravenous infusion [3]. Midazolam is a benzodiazepine and works by acting as a positive allosteric modulator of the $GABA_A$ receptor. Ketamine is a widely used drug in anaesthetic practise and, although it has a rich binding profile, its principal mode of action is as a NMDA receptor antagonist [4], whose endogenous ligand is the excitatory neurotransmitter glutamate. Together, $GABA_A$ and NMDA receptors are key players in shaping the activity of neuronal microcircuits. Thus, our experiment will provide complementary modifications of

excitation/inhibition balance in the brain which we will measure with EEG/fMRI [5]. Ketamine is a highly clinically relevant drug to understand given its new found action as an experimental antidepressant [6].

Resting-state measurements

Several resting-state studies have been conducted with separate EEG and fMRI recordings using similar drug interventions and at present the combined results are difficult to resolve across modalities. For ketamine, the existing EEG/MEG data consistently show enhanced gamma, reduced alpha and an onset burst of theta with different pharmacodynamics [7-10]. These studies also show that ketamine tends to reduce cortical connectivity. On the other hand fMRI studies have shown widespread BOLD increases in response to ketamine with some decreases localised to the subgenual cingulate (e.g. [11, 12]). fMRI connectivity studies on the other hand generally show widespread hyperconnectivity with ketamine (e.g.[13, 14]). Without simultaneous recording it is hard to make sense of these disparities. In particular, there is the concern that the changes in heart-rate, blood pressure and ventilation that occur with ketamine may affect existing results, as most studies have not attempted to control for these factors. With regards to midazolam, benzodiazepines are well known to enhance beta and reduce theta rhythms. In fMRI studies reduced default mode network (precuneus) activity has been found with midazolam infusion (Greicius et al; 2008). Confusingly, in a classic early EEG/fMRI study (Laufs et al.; 2003) beta band activity was found to positively correlate with precuneus activity. Simultaneous EEG/fMRI with midazolam/ketamine may help to resolve these inconsistencies

Task-based measurements

We will use two tasks that have been used several times in the literature to determine whether pharmacological modulation decouples the relationship between EEG and fMRI. These tasks will be the N-Back paradigm which probes working memory and the MID task which indexes reward anticipation. A single paper has examined the N-Back task in two participants and shown co-localisation of fMRI and EEG responses (theta band responses) [15]. An fMRI study has examined the N-Back task with the oral benzodiazepine lorazepam and shown impairments in task performance and a reduction in BOLD responses [16], while behaviourally ketamine impairs performance on the N-Back task [17]. A previous EEG/fMRI study of the MID task has shown relationships between the CNV response of the EEG with fMRI responses in the ventral striatum and supplementary motor area [18]. Ketamine has previously been shown to dampen reward anticipation responses in the ventral striatum measured with fMRI alone [19] while benzodiazepines are known to reduce the CNV [20]. Our study will therefore test whether the known simultaneous EEG/fMRI responses seen in these tasks become decoupled (or not) under pharmacological interventions.

5. AIMS

The aims of this study are largely exploratory. The data collected here will be one of the largest simultaneous EEG/fMRI studies using pharmacological interventions ever collected. This will enable extensive exploratory analysis of both task-based and resting-state brain imaging data.

6. LIST OF MEASURES

There are no clinical endpoints in this study. Brain-imaging, physiological and psychological measures that will be collected will include:

- Simultaneous EEG/fMRI measurements of resting-state brain activity.
- Simultaneous EEG/fMRI measurements of task-based brain activity.
- ASL measurements of resting-state brain activity.
- Physiological measures including: end tidal $C0_2$ and O_2, respiratory force and heart rate measured by ECG and pulse-oximetry.
- Psychological measures including visual analog scales for sedation / drug effects and the 5D-ASC.
- Cognitive measures including the NIH Toolbox Cognition battery.

7. STUDY DESIGN

The overall study design is a randomised, single-blinded, placebo-controlled crossover study of ketamine, placebo and midazolam in healthy volunteers. Following the giving of informed consent and the checking of eligibility; participants will be randomly assigned (without replacement) to one of six groups who will take part in the study days in the following orders: PKM PMK MPK MKP KMP KPM (where P = Placebo; M = Midazolam; K = Ketamine). With a sample size of 30 there will be 5 members in each of these groups. Starting on a separate day after the screening visit, participants will make three visits to CAMRI – the MRI research facility located within the Faculty of Medical and Health Sciences, Auckland University. A timeline for each study is provided in Table 1. Specific Scan details are provided in Table 2

Table 1: Timeline of Study Day Procedures for participants.

**Time**	**Procedure**
0:00 – 0:10	Greet and screen participant
0:10 – 1:10	EEG preparation, electrode digitsation and cannulation
1:10 – 1:30	Enter MRI Suite
1:30 – 2:30	MRI scan
2:30 – 3:30	Recovery
3:30 onwards	Discharge at discretion of investigators

Table 2: Scans to be performed.

**Duration*****	**Procedure**
1	Localiser scan
5	MPRAGE (Structural scan)
17	Resting-state (fMRI/EEG) (Drug infusion starts at 7 mins)
10	N-Back task (fMRI/EEG)
15	Reward Task (fMRI/EEG)
5	EEG Cap removal
8	PC-ASL
5	Breath hold task (FMRI) (Drug infusion ends)
66	**Total**

**** Indicative timings only. These will be finalised during pilot testing.*

Washout Period

The elimination half-life for midazolam is 1.5-2.5 hours and for ketamine 2.5 hours (see links in Section 15). Maximum total clearance time is therefore less than 24 hours (5 half-lives x 2.5). We will therefore employ a minimum inter-session interval of 48 hours in this study.

8. PARTICIPANT IDENTIFICATION

8.1. Study Participants

30 healthy male volunteers will be recruited to complete this study. These participants will be recruited from advertisements placed in local newspapers / noticeboards and social media. Only male participants will be recruited to avoid the well-known effects of the menstrual cycle on both the EEG and GABAergic system.

8.2. Inclusion Criteria

- Participant is willing and able to give informed consent for participation in the study.
- Male aged 18 years or above and less than 45 years old.
- In the Investigators' opinion, is able and willing to comply with all study requirements.

8.3. Exclusion Criteria

The participant may not enter the study if ANY of the following apply:

- Cardiovascular conditions including abnormal heart rate and blood pressure checked at screening.
- Participants who have participated in another research study involving an investigational product in the past 12 weeks.
- History of psychosis or personality disorder.
- Any unstable medical or neurologic condition.
- Substance abuse or dependence in previous 6 months.
- Any history of abuse of ketamine or benzodiazepenes.
- Contraindication to the use of ketamine/midazolam according to manufacturer guidelines.
- Body-weight <50kg or >120kg.
- BMI range 18.5 - 30
- Failure of the Health Questionnaire section of the "Anaesthesia assessment patient questionnaire" endorsed by the New Zealand Society of Anaethetists.
- Any other condition judged by the treating clinician as likely to impact on the ability of the

participant to complete the study.

- Regular use of any medication deemed to be contraindicating as judged by the attending study physicians (e.g. benxodiazepenes / sedatives / sleeping agents / NMDA antagonists).
- Inability to speak or read English.
- Contraindications for MRI scanning as per standard CAMRI SOPs.
- Needle phobia
- History of claustrophobia

8.4. Pilot participants

We will study up to 20 healthy pilot participants such that we can pilot our drug delivery, imaging and data collection procedures. Exclusion criteria will be as in 8.3. Inclusion criteria will be as per 8.2. These participants may only take part in parts of the main experiment.

8.5. Loss of participants

In the event that a participant withdraws from the study after being randomised they will be replaced with a new participant into the study until the desired sample size is obtained. The replacement subject will adapt the treatment order (see 7.).

8.6. Payment of participants

All participants will be paid $15 per hour pro rata for time they give to the study in the form of vouchers plus they will receive a bonus of up to $60 per session for successful performance of the MID task. Reasonable travel expenses will be paid either by reimbursement on production of receipts or paid for in advance by the investigators. Participants will receive an extra $50 for completing all three sessions.

9. STUDY PROCEDURES

9.1. Recruitment

An initial screening will be conducted by a researcher in response to phone or email contact made by the participant. Participants who pass initial screening will be given an appointment to visit, where they will have formal assessments completed to ensure they fulfill criteria to enter the study. The research team will not contact the participant's GP to inform them of their participation in the study.

9.2. Informed Consent

Written and verbal versions of the Participant Information Sheet and Informed Consent form will be presented to the participants detailing no less than: the exact nature of the study; what it will involve for the participant; the implications and constraints of the protocol; the known side effects and any risks involved in taking part. It will be clearly stated that the participant is free to withdraw from the study at any time for any reason without prejudice to future care, and with no obligation to give the reason for withdrawal.

The participant will be allowed as much time as wished to consider the information, and the opportunity to question the Investigator, their GP or other independent parties to decide whether they will participate in the study. Written Informed Consent will then be obtained by means of participant dated signature and dated signature of the person who presented and obtained the Informed Consent. The person who obtains consent will be an investigator in the study. A copy of the signed Informed Consent form will be given to the participant. The original signed form will be retained in the TMF.

Verbal consent will be reconfirmed at the start of each study day.

9.3. Randomisation and Blinding

One member of our research team will perform randomisation of participants to groups prior to study commencement and this will be set out in a randomisation schedule. During participant debriefing, participants will be asked to identify which session they thought was which and then informed as to the correct identification of sessions.

9.4. Discontinuation/Withdrawal of Participants from Study Treatment

Each participant has the right to withdraw from the study at any time. In addition, an Investigator may discontinue a participant from the study at any time if the Investigator considers it necessary for any reason including:

- Ineligibility (either arising during the study or retrospectively having been overlooked at screening)
- Significant protocol deviation
- Significant non-compliance with study requirements
- An adverse event which requires discontinuation of the study medication or results in inability to continue to comply with study procedures
- Withdrawal of consent
- Loss to follow up

The reason for withdrawal will be recorded in the CRF.

If the participant is withdrawn due to an adverse event, an Investigator will arrange for follow-up visits or telephone calls and any necessary treatment until the adverse event has resolved or stabilised.

9.5. Definition of End of Study

The end of study is the date of the last session of the last participant.

9.6. Schedule of Procedures

Procedures	Screening Visit	Visit 1	Visit 2	Visit 3
Informed consent	●			
Demographics	●			
Medical history	●			
Medical Examination	●			
Screening tests	●			
Eligibility assessment	●			
Cognitive/Psychological Testing	●			
Task training	●			
Randomisation	●			
Pre-scan preparation		●	●	●
Administration of study drugs		●	●	●
MRI		●	●	●
EEG		●	●	●
Peripheral Physiology		●	●	●
Blood sampling		●	●	●
Psychometric Questionnaires		●	●	●
Debrief				●

9.7. Summary of Procedures

Informed Consent, Demographics, Medical History, and medical examination

Signed consent forms will be kept in the TMF. Information regarding demographics, medical history, and medical examination will be documented in the CRF.

Screening Tests

All participants will provide a urine samples at the screening visit. A positive test for recreational drugs using a multi-panel screen will result in ineligibility. Results will be documented in the CRF.

Eligibility Assessment

Given the information supplied in the proceeding schedules the eligibility of the participant to take part in the study will be confirmed.

Cognitive/Psychological Testing

This will include the NIH Toolbox Cognition battery, The Eysenck Personality Questionnaire, The Spielperger Trait Anxiety Inventory, Becks Depression Inventory and the Schizotypal Personality Questionnaire.

Task training

Participants will receive training on the tasks to be performed in the scanner (N-Back Task / MID task).

Randomisation

See 9.3

Pre-scan preparation

This will include, MRI safety screening and changing into MR appropriate clothing, EEG electrode preparation and digitisation, further task training and insertion of an IV cannula.

Administration of Study Drugs

An intravenous line controlled by an infusion pump programmed by the supervising anaesthesiologist will deliver drugs. Heart-rate, oxygen saturation, respiratory force and end-tidal CO_2 levels will be continuously monitored during the administration of drugs. At the end of the scanning session heart rate and blood pressure will be monitored for one hour at 15 minute intervals by the supervising clinician.

MRI

MRI data will be acquired at the CAMRI located on the Grafton campus at The University of Auckland equipped with a 3T Siemens Skyra scanner using a 20 channel head coil. Sequences will include the

acquisition of localiser images, structural MRI (MPRAGE), functional MRI (GE-EPI) and Psuedo Continuous Arterial Spin labelling (PCASL). Precise scan parameters will be otained during pilot testing.

EEG

64-channel EEG data will be acquired simultaneous to the acquisition of functional MRI data. These data will be recorded using BrainAmp MR plus amplifiers and 64-channel Braincaps provided by Brain Products. A Brain Products SyncBox will be used to synchronise the acquisition of EEG and fMRI data.

Peripheral Physiology

Peripheral physiological measures will be measured on a Biopac MP150 system. Specific measures will include pulse plethsymograms, respiratory force, end-tidal O_2 and CO_2. Oxygen saturation will be monitored but not recorded.

Blood sampling

Blood samples of approximately 15 ml (3 teaspoons) will be obtained for laboratory tests including measurement of serum BDNF and ketamine derivatives. Sample will be taken pre-processed and sorted in -80°C freezers for subsequent analysis. Samples for genetic analysis will be obtained for post-hoc exploratory analysis.

Psychometric Questionnaires

Visual analog scales of drug effects and sedation will be recorded while participants in the scanner. Participants will complete the 5D-ASC retrospectively after the scanning session

Debrief

See 9.5

10. INVESTIGATIONAL MEDICINAL PRODUCTS (IMP)

10.1. Storage of Ketamine and Midazolam

Ketamine and midazolam will be stored at room temperature in the locked School of Pharmacy stores in the Faculty of Medical and Health Sciences, Auckland University. The School of Pharmacy holds the appropriate licenses to store these medications

10.2. Accountability of the Study Treatments

The attending clinician, who will be a registered medical practitioner in New Zealand, but not necessarily a member of the investigative team, will administer the treatments to be used. All administrations will be logged and signed for by the attending clinician and by one other member of the investigative team. These records will be held in the TMF.

11. SAFETY

11.1. Definitions

Adverse Event (AE)	Any untoward medical occurrence in a participant to whom a medicinal product has been administered, including occurrences which are not necessarily caused by or related to that product.
Adverse Reaction (AR)	An untoward and unintended response in a participant to an investigational medicinal product which is related to any dose administered to that participant.<br>The phrase "response to an investigational medicinal product" means that a causal relationship between a study medication and an AE is at least a reasonable possibility, i.e. the relationship cannot be ruled out.<br>All cases judged by either the reporting medically qualified professional or the Sponsor as having a reasonable suspected causal relationship to the study medication qualify as adverse reactions.
Serious Adverse Event (SAE)	A serious adverse event is any untoward medical occurrence that:<br>• results in death<br>• is life-threatening<br>• requires inpatient hospitalisation or prolongation of existing hospitalisation<br>• results in persistent or significant disability/incapacity
Serious Adverse Reaction (SAR)	An adverse event that is both serious and, in the opinion of the reporting Investigator, believed with reasonable probability to be due to one of the study treatments, based on the information provided.
Suspected Unexpected Serious Adverse Reaction (SUSAR)	A serious adverse reaction, the nature and severity of which is not consistent with the information about the medicinal product in question.

11.2. Causality

The relationship of each adverse event to the study medication must be determined by a medically qualified individual according to the following definitions:

Related: The adverse event follows a reasonable temporal sequence from study medication administration. It cannot reasonably be attributed to any other cause.

Not Related: The adverse event is probably produced by the participant's clinical state or by other modes of therapy administered to the participant.

11.3. Procedures for Recording Adverse Events

All AEs occurring during the study will be recorded on the CRF, whether or not attributed to study medication. The following information will be recorded: description, date of onset and end date, severity, assessment of relatedness to study medication and action taken. Follow-up information should be provided as necessary. Severity of events will be assessed on the following scale: 1 = mild, 2 = moderate, 3 = serious.

11.4. Reporting Procedures for Serious Adverse Events

A complete report of All SAEs will be reported to the Safety Monitoring Committee of the study within 24 hours. A report will also be sent to the Centre for Adverse Reactions Monitoring following MedSafe guidelines (https://nzphvc.otago.ac.nz/carm/).

11.5. Safety Monitoring Committee

The Safety Monitoring Committee for this study will comprise of two consultant anaesthetists.

Dr Douglas Campbell
Consultant Anaethetist
Auckland City Hospital
Auckland District Health Board

Dr Tim Short
Consultant Anaethetist
Auckland City Hospital
Auckland District Health Board

In the unlikely event of an SAE being reported, the Safety Monitoring Committee may decide to suspend the study or request suspension until the research protocol is appropriately revised.

11.6 Safety Protocols

a) In case of unexpected adverse effects participants will be treated onsite by the attending clinician. The study site is located across the road from the Auckland City Hospital and is connected by an underground tunnel. A SOP exists at CAMRI for calling Auckland City Hospital emergency teams.
b) Should participants experience adverse events such as psychotomimetic reactions, extreme claustrophobia or panic attacks while they are on site, the supervising clinician will administer an appropriate course of treatment.
c) In case of unexpected adverse effects, the study site is located across the road from the Auckland City Hospital Emergency Department and the estimated time of transport is approximately 5-10 minutes.

12. DATA ANALYSIS AND STATISTICS

12.1. Power calculations

Given the exploratory nature of the study and the unknown effects sizes, estimates of required sample size cannot be reliably provided. As an alternative, a sensitivity analysis conducted in G*Power 3.1 [21] demonstrates that with a sample size of 30, a significance-level of $\alpha = 0.05$, $\beta = 0.8$ that we will be sensitive to effect sizes of $d > 0.465$. Heuristically, this sample size is larger than normally seen in this kind of brain imaging research.

12.2. Analytical Methods

For data analysis we will use a combination of standard brain imaging software packages, analysis toolboxes and custom scripts. Some software packages that will be used include BrainVision Analyzer®, the FSL software library and the Fieldtrip toolbox for MATLAB®. For first-pass analysis of EEG data, these will be gradient corrected and the ballistocardiogram artifact removed using BrainVision Analyzer software. Data quality will be inspected using fast fourier transforms. For first-pass analysis of fMRI data a standard pipeline in FSL (http://fsl.fmrib.ox.ac.uk/fsl/fslwiki/) will be used; this will include for example, motion correction using MCFLIRT, non-brain removal using BET, spatial smoothing using a Gaussian kernel of FWHM 5mm, grand-mean intensity normalisation of the entire 4D dataset by a single multiplicative factor and highpass temporal filtering.

13. DATA MANAGEMENT

13.1. Access to Data

Direct access will be granted to authorised representatives from the host institution and the regulatory authorities to permit study-related monitoring, audits and inspections.

13.2. Data Recording and Record Keeping

A unique study specific number will be used to identify participants in any database or electronic file. The name and any other identifying detail will NOT be included in any study data electronic file. On all study-specific documents, other than the signed consent and page one of the CRF (separately filed), the participant will be referred to only by the study participant code, not by name. Data will be held for a period of 15 years from the completion of the study.

13.3 Electronic Data Storage

After data is collected it will be uploaded to University of Auckland IT servers. Access to server folders holding the data will be password protected and limited to members of the research team. All computers on this network are subject to University of Auckland IT policies and procedures

(https://www.auckland.ac.nz/en/about/the-university/how-university-works/policy-and-administration/computing.html). Data is stored in multiple locations with off-site backup copies regularly maintained.

14. STUDY STEERING COMMITTEE

The role of the Study Steering Committee (SSC) is to provide overall supervision of the study. The SSC will be comprised of all the investigators of this study. In particular, the SSC will collaboratively develop and approve the final protocol; oversee progress of the study, adherence to the protocol, participant safety and consideration of new information; and be responsible for publication and dissemination. The SSC must be in agreement with the final protocol and, throughout the study, will take responsibility for:

- major decisions such as a need to change the protocol for any reason.
- monitoring and supervising the progress of the study.
- reviewing relevant information from other sources.

15. ETHICAL AND REGULATORY CONSIDERATIONS

15.1. Declaration of Helsinki

The Investigator will ensure that this study is conducted in accordance with the principles of the Declaration of Helsinki (2008).

15.2. ICH Guidelines for Good Clinical Practice

The Investigator will ensure that this study is conducted in conformity with relevant regulations and with the ICH Guidelines for Good Clinical Practice.

15.3. Approvals

The protocol, informed consent form, participant information sheet and any proposed advertising material will be submitted to HDEC for written approval. No research procedures will be commenced until all written approvals are obtained.

15.4. Reporting

The PI shall submit an End of Study notification to HDEC.

15.5. Participant Confidentiality

The study staff will ensure that the participants' confidentiality is maintained. Only an ID number on the CRF and any electronic database will identify participants. All identifiable documents will be stored securely and only accessible by study staff and authorised personnel.

15.6. Additional Ethical Considerations

Human tissue will be extracted stored and destroyed in compliance with the human tissue act and with appropriate cultural sensitivity.

Cultural sensitivity - note the section about Maori participants in the PIS. This study follows guidelines outlined in the Guidelines for Researchers on Health Research Involving Maori (HRC, 2010) and the guidelines on Maori responsiveness outlined on the Auckland University Faculty of Medical and health Sciences website. The Principal Investigator has consulted several times with Dr Helen Wihongi (Waitemata and Auckland DHB Maori Research Advisor) regarding these issues.

Driving – Participants will be advised not to drive on either of the study days, as their ability to do so could be impaired. It will be confirmed that no participants have driven to the research facility on the day of the study. We will recommend to participants that a family member or friend picks them up after each study and visitor parking is available at the study site. Alternatively taxi transportation will be arranged for volunteers.

Potential Adverse Effects – These are summarised in the Medsafe datasheets for ketamine (www.medsafe.govt.nz/profs/datasheet/k/ketamineinf.pdf) and midazolam (http://www.medsafe.govt.nz/profs/datasheet/m/MidazolaminjPfizer.pdf). As both medications are already approved medicines there is minimal chance of adverse effects given the screening procedures to be implemented. Members of our research team have extensive clinical experience in the administration of ketamine and midazolam (anaesthetists and registrar anaesthetists). Appropriate monitoring equipment will be used during drug administration and resuscitation facilities will be available.

Confidentiality - Will be maintained through use of non-identifiable study identifiers used on all collected data (see study design section).

Use of controlled substances - No controlled substance (ketamine / midazolam) will leave the Grafton campus of the University of Auckland.

Addiction potential – Both ketamine and midazolam have a moderate addiction potential similar quantitatively to alcohol [22]. Dependence usually occurs after repeated administrations and is extremely unlikely after the single dose schedule to be administered in this protocol. As a precaution participants will be screened and excluded for recreational drug use and excluded for prior substance dependence.

16. FUNDING

This study is jointly funded by: a Rutherford Discovery Fellowship awarded by the Royal Society of New Zealand to Dr Suresh Muthukumaraswamy, an investigator initiated grant from F Hoffman La Roche Ltd and a University of Auckland Doctoral Scholarship.

17. REFERENCES

1. Fink, M., *Remembering the lost neuroscience of pharmaco-EEG.* Acta Psychiatr Scand, 2010. **121**(3): p. 161-73.
2. Iannetti, G.D. and R.G. Wise, *BOLD functional MRI in disease and pharmacological studies: room for improvement?* Magn Reson Imaging, 2007. **25**(6): p. 978-88.
3. Clements, J.A. and W.S. Nimmo, *Pharmacokinetics and analgesic effect of ketamine in man.* Br J Anaesth, 1981. **53**(1): p. 27-30.
4. Stahl, S.M., *Mechanism of action of ketamine.* CNS Spectr, 2013. **18**(4): p. 171-4.
5. Logothetis, N.K., *What we can do and what we cannot do with fMRI.* Nature, 2008. **453**(7197): p. 869-878.
6. Duman, R.S. and G.K. Aghajanian, *Synaptic dysfunction in depression: potential therapeutic targets.* Science, 2012. **338**(6103): p. 68-72.
7. Kochs, E., et al., *Analgesic efficacy of low-dose ketamine. Somatosensory-evoked responses in relation to subjective pain ratings.* Anesthesiology, 1996. **85**(2): p. 304-14.
8. Lee, U., et al., *Disruption of frontal-parietal communication by ketamine, propofol, and sevoflurane.* Anesthesiology, 2013. **118**(6): p. 1264-75.
9. Muthukumaraswamy, S.D., et al., *Evidence that subanaesthetic doses of ketamine cause sustained disruptions of NMDA and AMPA-mediated frontoparietal connectivity in humans.* Journal of Neuroscience, 2015. **in press**.
10. Rivolta, D., et al., *Ketamine Dysregulates the Amplitude and Connectivity of High-Frequency Oscillations in Cortical-Subcortical Networks in Humans: Evidence From Resting-State Magnetoencephalography-Recordings.* Schizophr Bull, 2015. **41**(5): p. 1105-14.
11. De Simoni, S., et al., *Test-retest reliability of the BOLD pharmacological MRI response to ketamine in healthy volunteers.* Neuroimage, 2013. **64**: p. 75-90.
12. Deakin, J.F., et al., *Glutamate and the neural basis of the subjective effects of ketamine: a pharmaco-magnetic resonance imaging study.* Arch Gen Psychiatry, 2008. **65**(2): p. 154-64.
13. Driesen, N.R., et al., *Relationship of resting brain hyperconnectivity and schizophrenia-like symptoms produced by the NMDA receptor antagonist ketamine in humans.* Mol Psychiatry, 2013. **18**(11): p. 1199-204.
14. Gass, N., et al., *Sub-anesthetic ketamine modulates intrinsic BOLD connectivity within the hippocampal-prefrontal circuit in the rat.* Neuropsychopharmacology, 2014. **39**(4): p. 895-906.
15. Esposito, F., et al., *Distributed analysis of simultaneous EEG-fMRI time-series: modeling and interpretation issues.* Magn Reson Imaging, 2009. **27**(8): p. 1120-30.
16. Menzies, L., et al., *Effects of gamma-aminobutyric acid-modulating drugs on working memory and brain function in patients with schizophrenia.* Arch Gen Psychiatry, 2007. **64**(2): p. 156-67.
17. Morgan, C.J., et al., *Acute effects of ketamine on memory systems and psychotic symptoms in healthy volunteers.* Neuropsychopharmacology, 2004. **29**(1): p. 208-18.

18. Plichta, M.M., et al., *Simultaneous EEG and fMRI reveals a causally connected subcortical-cortical network during reward anticipation.* J Neurosci, 2013. **33**(36): p. 14526-33.
19. Francois, J., et al., *Ketamine Suppresses the Ventral Striatal Response to Reward Anticipation: A Cross-Species Translational Neuroimaging Study.* Neuropsychopharmacology, 2015.
20. Rockstroh, B., et al., *Effects of the anticonvulsant benzodiazepine clonazepam on event-related brain potentials in humans.* Electroencephalogr Clin Neurophysiol, 1991. **78**(2): p. 142-9.
21. Faul, F., et al., *G*Power 3: A flexible statistical power analysis program for the social, behavioral, and biomedical sciences.* Behavior Research Methods, 2007. **39**(2): p. 175-191.
22. Nutt, D., et al., *Development of a rational scale to assess the harm of drugs of potential misuse.* Lancet, 2007. **369**(9566): p. 1047-53.
