# Supplementary material for: Local activity alterations in individuals with autism correlate with neurotransmitter properties and ketamine-induced brain changes
Source: Nat Commun. 2025 Sep 9;16:8248. doi: 10.1038/s41467-025-63857-6 (PMC12420803; doi:10.1038/s41467-025-63857-6)
Supplement: Supplementary file 2 — Reporting Summary [file 41467_2025_63857_MOESM2_ESM.pdf]

Corresponding author(s): Prof. Juergen Dukart

Last updated by author(s): Aug 20, 2025

Reporting Summary

Nature Portfolio wishes to improve the reproducibility of the work that we publish. This form provides structure for consistency and transparency in reporting. For further information on Nature Portfolio policies, see our Editorial Policies and the Editorial Policy Checklist.

Statistics

For all statistical analyses, confirm that the following items are present in the figure legend, table legend, main text, or Methods section.

n/a	Confirmed	
☐	☒	The exact sample size (*n*) for each experimental group/condition, given as a discrete number and unit of measurement
☐	☒	A statement on whether measurements were taken from distinct samples or whether the same sample was measured repeatedly
☐	☒	The statistical test(s) used AND whether they are one- or two-sided<br>*Only common tests should be described solely by name; describe more complex techniques in the Methods section.*
☐	☒	A description of all covariates tested
☐	☒	A description of any assumptions or corrections, such as tests of normality and adjustment for multiple comparisons
☐	☒	A full description of the statistical parameters including central tendency (e.g. means) or other basic estimates (e.g. regression coefficient) AND variation (e.g. standard deviation) or associated estimates of uncertainty (e.g. confidence intervals)
☐	☒	For null hypothesis testing, the test statistic (e.g. F, t, r) with confidence intervals, effect sizes, degrees of freedom and P value noted<br>*Give P values as exact values whenever suitable.*
☐	☒	For Bayesian analysis, information on the choice of priors and Markov chain Monte Carlo settings
☐	☒	For hierarchical and complex designs, identification of the appropriate level for tests and full reporting of outcomes
☐	☒	Estimates of effect sizes (e.g. Cohen's d, Pearson's r), indicating how they were calculated

Our web collection on statistics for biologists contains articles on many of the points above.

Software and code

Policy information about availability of computer code

Data collection	No software was used for data collection.
Data analysis	SPM12, Matlab (v2022b), CONN (v22a), JuSpace (v1.5), SPSS (version 27).

For manuscripts utilizing custom algorithms or software that are central to the research but not yet described in published literature, software must be made available to editors and reviewers. We strongly encourage code deposition in a community repository (e.g. GitHub). See the Nature Portfolio guidelines for submitting code & software for further information.

Data

Policy information about availability of data

All manuscripts must include a data availability statement. This statement should provide the following information, where applicable:

- Accession codes, unique identifiers, or web links for publicly available datasets
- A description of any restrictions on data availability
- For clinical datasets or third party data, please ensure that the statement adheres to our policy

The pharmacological data generated in this study are available under restricted access due to ethical approval and informed consent restrictions; access can be obtained by contacting the corresponding author (Prof. Juergen Dukart, j.dukart@fz-juelich.de) and providing a signed data use agreement and institutional ethics committee approval. Raw pharmacological data are protected and are not available due to data privacy laws. The processed pharmacological data are available in the Source Data files accompanying this paper and contain only aggregated summary values that do not permit identification of individual participants.
The autism neuroimaging data used in this study are available in the Autism Brain Imaging Data Exchange (ABIDE) repository (https://fcon_1000.projects.nitrc.org/

indi/abide/; doi:10.1038/mp.2013.78). The original ABIDE data are de-identified but subject to the repository's data use terms, and cannot be redistributed directly. The processed ABIDE data generated in this study are available in the Source Data files accompanying this paper and contain only aggregated and derived metrics that do not permit identification of individual participants.
Source data, results from whole-brain voxel-wise analyses, and thresholded result maps have been deposited in Zenodo (https://doi.org/10.5281/zenodo.16904492).

Research involving human participants, their data, or biological material

Policy information about studies with human participants or human data. See also policy information about sex, gender (identity/presentation), and sexual orientation and race, ethnicity and racism.

Reporting on sex and gender	We included autism data from both male and female participants, with a notably higher proportion of males (ABIDE1: 84.7%; ABIDE2: 77.8%). To address this imbalance, we applied sex as a covariate in voxel-wise comparisons of functional activity using the CONN toolbox. For the pharmacological dataset, only male participants were included to avoid variability in GABA levels associated with the menstrual cycle.
Reporting on race, ethnicity, or other socially relevant groupings	We did not report race, ethnicity or other socially relevant variables in our study.
Population characteristics	Age, sex, site and full-scale IQ were included as covariates in all analyses, along with head motion parameters. Participants with autism ranged in age from 5 to 64 years, while participants in the pharmacological dataset were aged 19 to 37 years. Subjects with intellectual disability (IQ ≤ 70) were excluded to avoid potential confounding effects on functional activity. Clinical symptom severity in the autism cohort was assessed using the Autism Diagnostic Observation Schedule (ADOS) for correlation analyses. For detailed information on recruitment procedures and diagnostic criteria, please refer to the ABIDE project website: https://fcon_1000.projects.nitrc.org/indi/abide.
Recruitment	Participants within the pharmacological fMRI dataset were recruited via advertisement on campus flyers and University of Auckland websites.
Ethics oversight	Written and informed consent was obtained from all participants, and the study was approved by the local Ethics Committee of the University of Auckland (Central Health and Disability Ethics Committee, Ref: 15/CEN/254). The trial is registered at https://www.anzctr.org.au/Trial/Registration/TrialReview.aspx?id=370230. The multicenter ABIDE dataset was collected with approval from the respective local ethics committees at each contributing site.

Note that full information on the approval of the study protocol must also be provided in the manuscript.

Field-specific reporting

Please select the one below that is the best fit for your research. If you are not sure, read the appropriate sections before making your selection.

[x] Life sciences [ ] Behavioural & social sciences [ ] Ecological, evolutionary & environmental sciences

For a reference copy of the document with all sections, see nature.com/documents/nr-reporting-summary-flat.pdf

Life sciences study design

All studies must disclose on these points even when the disclosure is negative.

Sample size	We used publicly available data from ABIDE to leverage large, well-characterized multicenter cohorts of individuals with and without autism. ABIDE1 included N = 878 subjects (405 with autism), and ABIDE2 included N = 869 subjects (395 with autism). These two independent datasets allowed us to adopt an exploration-and-replication approach, increasing the robustness and generalizability of our findings. The pharmacological dataset included N = 30 participants (n = 27 after quality control), which is consistent with sample sizes typically used in controlled pharmacological fMRI studies and sufficient to detect within-subject effects in a three-way crossover design. Together, the combination of large-scale public datasets for autism and a well-controlled pharmacological dataset provides sufficient statistical power to address the primary research questions regarding LCOR alterations and their neurochemical co-localization.
Data exclusions	Autism data were excluded in cases of intellectual disability (IQ < 71), excessive head motion during image acquisition, missing data, or preprocessing failure of imaging data (ABIDE1: n = 234; ABIDE2: n = 245). For the pharmacological dataset, three participants were excluded due to image quality control issues.
Replication	We adopted an exploration-and-replication approach for the autism data. Significant LCOR alterations and neurotransmitter co-localizations were first tested in ABIDE1 and subsequently assessed for replication in ABIDE2. Findings were considered robust if they reached statistical significance in both datasets ($p < .05$). For the pharmacological dataset, each participant underwent a three-way crossover design with ketamine, midazolam, and placebo conditions, allowing within-subject replication of the fMRI measurements across the three sessions.<br><br>Increased LCOR in autism was successfully replicated, along with co-localization with dopaminergic (D1, D2, DAT), glutamatergic (NMDA, mGluR5), GABAergic (GABAa), and cholinergic (VAChT) neurotransmission. Whole-brain LCOR changes following ketamine administration showed significant associations with autism-related LCOR differences in both ABIDE1 and ABIDE2. The neurochemical co-localization profile of ketamine-induced LCOR changes was significantly associated with the autism co-localization profile observed in ABIDE2, while the association with ABIDE1 reached only a trend level, potentially due to the sociodemographic and clinical heterogeneity in the autism samples.
Randomization	Participants were divided into an autism group and a typically developing (TD) group based on clinical interviews and diagnoses. In the autism

	data, analyses controlled for age, sex, full-scale IQ, site, and head motion parameters. Participants in the pharmacological dataset were scanned in a three-way crossover design, receiving ketamine, midazolam, or placebo. A random-number generator was used to assign participants to six distinct condition-order groups. In analyses of the pharmacological dataset, age and head motion were included as covariates.
Blinding	Participants within the pharmacological dataset were scanned single-blinded.

Reporting for specific materials, systems and methods

We require information from authors about some types of materials, experimental systems and methods used in many studies. Here, indicate whether each material, system or method listed is relevant to your study. If you are not sure if a list item applies to your research, read the appropriate section before selecting a response.

Materials & experimental systems

n/a	Involved in the study
☒	☐ Antibodies
☒	☐ Eukaryotic cell lines
☒	☐ Palaeontology and archaeology
☒	☐ Animals and other organisms
☐	☒ Clinical data
☒	☐ Dual use research of concern
☒	☐ Plants

Methods

n/a	Involved in the study
☒	☐ ChIP-seq
☒	☐ Flow cytometry
☐	☒ MRI-based neuroimaging

Clinical data

Policy information about clinical studies

All manuscripts should comply with the ICMJE guidelines for publication of clinical research and a completed CONSORT checklist must be included with all submissions.

Clinical trial registration	https://www.anzctr.org.au/Trial/Registration/TrialReview.aspx?id=370230<br>Trial registration number: ACTRN12616000281493
Study protocol	The study protocol has not been formally published. The trial is registered at https://www.anzctr.org.au/Trial/Registration/TrialReview.aspx?id=370230, and the full study protocol can be made available upon reasonable request to the corresponding authors.
Data collection	Data was collected at the University of Auckland, data collection was between 24/03/2016 and 15/12/2016.
Outcomes	Primary outcomes were power spectral density of the EEG, and secondary outcomes were functional activity measured with functional magnetic resonance imaging (fMRI), both assessed 0–10 minutes post-administration. Please note that these outcomes have been reported previously (Forsyth et al., 2018; Forsyth et al., 2020); the present analyses focus exclusively on the fMRI data.

Plants

Seed stocks	None.
Novel plant genotypes	None.
Authentication	None.

Magnetic resonance imaging

Experimental design

Design type	We collected resting-state functional MRI data.
Design specifications	See https://fcon_1000.projects.nitrc.org/indi/abide and Supplementary Tables S17-18 for varying recruitment criteria of ABIDE data across sites. For the pharmacological dataset, each participants was scanned pre and post administration for the three blocks respectively (ketamine, midazolam, placebo). Acquisition time was 7 minutes predrug and 9 minutes postdrug. A 3 day minimum washout between sessions was employed.

Behavioral performance measures | No behavioral performance measures were acquired.

Acquisition

Imaging type(s): Functional, Structural

Field strength: 3T

Sequence & imaging parameters: For the pharmacological dataset: For structural imaging, a 3D magnetization-prepared rapid gradient-echo (3D-MPRAGE) scan [echo time (TE) = 3.42 ms; repetition time (TR) = 2100 ms; FOV = 256 mm2; flip angle 9°; 192 slices; slice thickness = 2 mm; voxel size = 1x1x1 mm] was acquired. Additionally, 246 volumes of BOLD rs-fMRI data were obtained using a T2*-weighted echo planar imaging (EPI) sequence (TE = 27 ms; TR = 2200 ms; flip angle 79°; 30 interleaved 3 mm slices; voxel size = 3x3x3 mm).
See https://fcon_1000.projects.nitrc.org/indi/abide and Supplementary Tables S17-18 for varying sequence and imaging parameters of ASD data across sites.

Area of acquisition: A whole-brain scan was acquired.

Diffusion MRI ☐ Used ☒ Not used

Preprocessing

Preprocessing software: SPM12, Matlab (v2022b), CONN (v22a) - Functional images were corrected for head motion and distortions (realign and unwarp). Smoothing was applied using a 6 mm full-width at half maximum (FWHM) Gaussian kernel.

Normalization: Functional images were non-linear spatially normalized into standard MNI space and resampled to a resolution of 2 mm3 isotropic voxels.

Normalization template: Standard MNI space with 180x216x180 mm bounding box.

Noise and artifact removal: Mean white matter, grey matter and cerebrospinal fluid signals, as well as 24 motion parameters were regressed out before computing the voxel-based measures. Motion parameters were used to identify data to be excluded due to excessive head movement (translation > 3 mm or rotation > 3°).

Volume censoring: Participants were excluded in case of excessive head movement (translation > 3 mm or rotation > 3°).

Statistical modeling & inference

Model type and settings: We used mass univariate voxel-wise t-contrasts between subjects with autism and TD controls.

Effect(s) tested: We tested the effect of the autism diagnosis on resting-state local functional activity (relative to TD controls). We did not use ANOVA or factorial designs.

Specify type of analysis: ☒ Whole brain ☐ ROI-based ☐ Both

Statistic type for inference (See Eklund et al. 2016): We used a family-wise error (FWE) threshold of p < 0.05 combined with an exact permutation-based (1000 permutations) threshold free cluster enhancement (TFCE, p < 0.05).

Correction: We used FWE combinded with TFCE to control for multiple testing.

Models & analysis

n/a	Involved in the study
☐	☒ Functional and/or effective connectivity
☒	☐ Graph analysis
☒	☐ Multivariate modeling or predictive analysis

Functional and/or effective connectivity: We used local synchronization (local correlation or LCOR) by computing Pearson correlations at each voxel.
